# The role of *E. maritimum* (L.) in the dune pollination network of the Balearic Islands

**DOI:** 10.1002/ece3.9164

**Published:** 2022-08-04

**Authors:** Iván Cortés‐Fernández, Marcello Dante Cerrato, Arnau Ribas‐Serra, Xavier Canyelles Ferrà, Lorenzo Gil‐Vives

**Affiliations:** ^1^ Interdisciplinary Ecology Group Universitat de les Illes Baleares, Carretera de Valldemossa Palma de Mallorca Spain

**Keywords:** connectance, dune, *Eryngium maritimum*, pollination network, resilience

## Abstract

*Eryngium maritimum* L. (Apiaceae) is a geophyte that inhabits in the dunes of the Mediterranean and Atlantic. Although it is a highly entomophilous species, there is little literature on its pollinator assemblage. The aim of this study is to analyze the role played by *E. maritimum* in the dune pollination network of the Balearic Islands, where there is an intense anthropogenic impact in its habitat. For this purpose, two populations located in the North and South of Mallorca were chosen, in which diurnal transects were carried out to observe and capture pollinators on 15 plant species during the anthesis period of *E. maritimum*. The flowering period of 10 plant species flowering at the same period than *E. maritimum* was analyzed to identify periods of competition. A total of 71 pollinator species were found, belonging to 30 different families. *Eryngium maritimum* is a strongly generalist species, with a total of 45 pollinator species. Two new species, *Odice blandula* and *Leucospis gigas*, were found for the first time in Mallorca. In terms of pollinators, *Teucrium dunense* and *Helichrysum stoechas* are the most similar species to *E. maritimum*. However, analysis of phenology suggests that these three species have been able to decouple their blooms to avoid competition. The present study shows that *E. maritimum* plays an important role in the dune pollination network, being its anthesis located at the end of the dune flowering season, when there are no functionally similar species in flower.

## Graphical abstract

*Eryngium maritimum* (L.) plays a key role in the dune pollination network of the Balearic islands. It behaves as an opportunistic species and is visited by more than 80 different species. Its conservation is vital to maintain the complex ecological network of this coastal habitat.

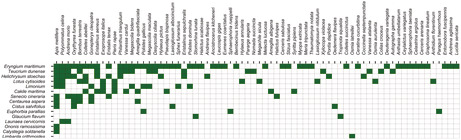

## 1 INTRODUCTION

Pollination is considered as one of the most crucial plant–animal interactions, influencing on dynamics and diversity of plant communities (Fantinato, Del Vecchio, Giovanetti, et al., 2018). Widespread declines in pollinators had led to a concern about a global pollination crisis (Burkle et al., 2013; Tylianakis, 2013). By reducing pollinator availability and nesting sites due to habitat modifications, cross‐pollination levels can be modified influencing plant fruit and seed production (Traveset et al., 2018; Vanbergen et al., 2014). At the same time, anthropization is jeopardizing the conservation of ecosystems and the ability to resist future environmental changes (MacDougall et al., 2013). Among ecosystems, coastal habitats, such as seashores and dune ridges, are considered some of the most threatened habitats (Gigante et al., 2018), due to habitat loss (Coverdale et al., 2013), global warming (Culbertson et al., 2009) and coastal salination due to an increased sea‐level (Chu‐Agor et al., 2011). Concretely, coastal dune ecosystems are a hotspot for specialized pollinator species (Cane, 1991), displaying highly specialized species and interactions higher risks of extinction (Aizen et al., 2012; Burkle et al., 2013). Understanding plant–pollinator interactions is vital to give light to coevolutionary processes in highly diverse communities (Bascompte & Jordano, 2007) and to evaluate the maintenance of ecosystem's resilience over time (Fantinato et al., 2019). So, pollinators are used as a bioindicator species as the decline of their populations are strongly associated with anthropogenic influence (Biesmeijer et al., 2006). At the same time, some attributes of the pollination network (selectiveness, nestedness, connectance) have an ecological meaning in the assessment of habitat resilience to various forms of disturbances (Fantinato et al., 2019; Lázaro et al., 2016; Traveset et al., 2018).

Ecological indicators enable the analysis of complex systems processed in a reliable way (Dale & Beyeler, 2001). Selectiveness or specialization is defined as the number of partners, or links, of a species (Blüthgen et al., 2006). Specialist species are usually the first to go extinct from a network (Henle et al., 2004), Connectance (or link density) is the most common way to characterize specialization and is calculated as the proportion of the observed interactions to all possible interactions (Olesen & Jordano, 2002). It is considered as a useful metric to analyze functional redundancy of interactions, which is related with resilience, due to its ease of calculation (Tylianakis et al., 2010). On the other hand, the interactions in a network are said to be nested when the species interacting with specialists are a proper subset of the species interacting with generalists (Tylianakis et al., 2010). The ecological implication of nestedness is that, if an species goes extinct and the network is nested, the remaining species will have others with which to interact, providing a buffer to secondary extinctions (Fortuna & Bascompte, 2006; Tylianakis et al., 2010). Compartmentalization is interpreted as a subset of an interaction network which tend to interact frequently with another, but little with the species outside of the compartment (Tylianakis et al., 2010). Compartmentalization may be caused by coevolution, and it is considered that increases stability of networks (Krause et al., 2003). Highly connected species within a compartment are considered as “module hubs”, while species interacting with various compartments are considered as connectors (Olesen & Jordano, 2002). In terms of conservation, the extinction of module hubs and connectors is related with cascading extinctions across compartments (Olesen et al., 2007; Tylianakis et al., 2010).

*Eryngium maritimum* L. is a geophyte from the Apiaceae family that inhabits in sand dunes of the Atlantic and Mediterranean coasts (Isermann & Rooney, 2014), being a diagnostic species of mobile dunes (Marcenò & Jiménez‐Alfaro, 2017). Each individual produces one flowering stalk, from which multiple capituliform inflorescences (5–40) emerge in a dichasial disposition (Cortés‐Fernández, Cerrato, Ribas‐Serra, & Gil Vives, 2022). Flowers per capitula are numerous (25–50), hermaphrodite, with nectaries at the base, while stamens are prominent, purplish to bluish (Isermann & Rooney, 2014). Its role in the coastal pollination network has never been assessed, and its pollinators are unstudied, with only a few studies that give light to some of its pollinators (Gil, 1994) and most of them carried out in Northern European populations (Fitter & Peat, 1994; Hegi, 1935; Westrich, 2001; Zanella et al., 2009), where it is considered as a highly‐threatened species (Aviziene et al., 2008; van der Maarel & van der Maarel‐Versluys, 1996). In Northern populations, it displays low fruit and seed set production, and conversely, Balearic populations exhibit high fruit and seed set, with low levels of incompatibility (Cortés‐Fernández et al., 2021). These differences could be related to pollinators but, to validate this hypothesis, firstly it is compulsory to understand how the species behaves in Mediterranean populations, where its populations exhibit a good conservation status.

In the Balearic Islands, *E. maritimum* develops optimally in embryonic and white dunes, where perennial grasses are not dominant (Llorens et al., 2021). The dune systems of the Balearic Islands are exposed to intense levels of anthropization, mainly due to the touristic pressure (García & Servera, 2003), but also to invasive species introduction (Hulme et al., 2008; Moragues & Traveset, 2005), and will be specially affected by coastal retreat (Enríquez et al., 2017). In Mallorca, it coinhabits with a great variety of plant species which are strongly pollinator‐dependent, including members of the Lamiaceae (*Teucrium dunense* Sennen), Leguminosae (*Lotus cytisoides* L.), Asteraceae (*Helichrysum stoechas* (L.) Moench) and Papaveraceae (*Glaucium flavum* Crantz). The best approaches to give light to the dune pollination networks of the Balearic Islands have been carried out in two locations: Son Bosc and Cala Mesquida. Son Bosc is a fixed dune of the North of Mallorca (Castro‐Urgal & Traveset, 2014; Lázaro et al., 2020; Traveset et al., 2017; Tur et al., 2013), which displays a substantially differential floral diversity than where *E. maritimum* optimally inhabits (Marcenò & Jiménez‐Alfaro, 2017). On the other hand, Cala Mesquida is the studied location more representative of the optimal habitat of *E. maritimum* but in the only study available in this area (Castro‐Urgal & Traveset, 2014), the specific role of *E. maritimum* is not analyzed.

The main objective of the present study is to understand which are the pollinators of *E. maritimum* and what is its specific role in the dune pollination network of the Balearic Islands. The main hypothesis is that *E. maritimum* is visited by a great number of pollinators, as attending to previous studies the capacity of the species to self‐fertilize is low, as well as its anemophily, which suggests that the species is strongly entomophilous (Cortés‐Fernández, Cerrato, Ribas‐Serra, & Gil Vives, 2022). This study will give light to the ecology of the species in the Balearic Islands, continuing a series of studies which analyzed its reproductive biology, germination and salinity tolerance in this area (Cortés‐Fernández et al., 2021; Cortés‐Fernández, Cerrato, Ribas‐Serra, & Gil, 2022; Cortés‐Fernández, Cerrato, Ribas‐Serra, & Gil Vives, 2022).

## 2 MATERIAL AND METHODS

### 2.1 Study area

**FIGURE 1:**
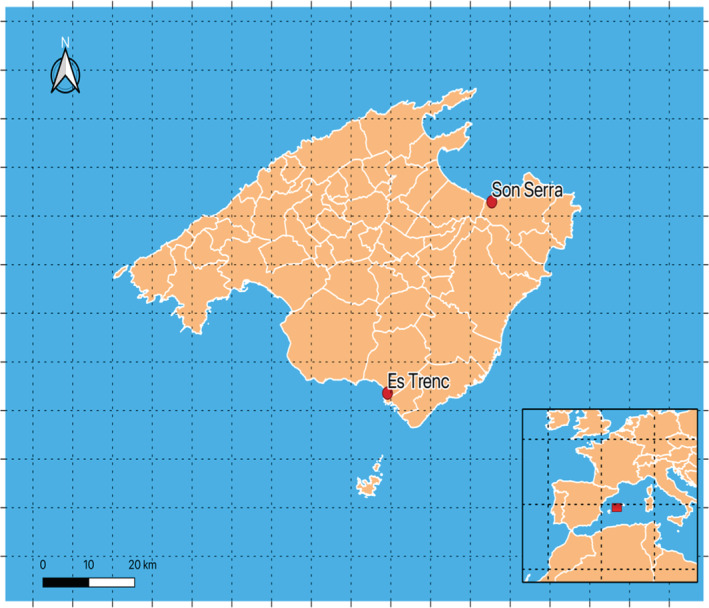
Map of the studied areas. Red points indicated the sampling areas, each one divided in 3 transects of 50 m separated by 100 m.

Pollinator surveys were carried out in two different coastal dunes of Mallorca (Balearic Islands, Spain), one located in the North and one in the South of the island (Figure 1). First sampling area was located in Son Serra de Marina (SS, 39.7309 N, 3.2382 E), in the North of the island. Although the area is relatively well‐conserved compared with other areas of the island, it suffers from severe anthropogenic impact mainly due to tourist pressure, overall in the drift line zone. On the other hand, the other sampling area was located in Es Trenc (ET, 39.3382 N, 2.9903 E), in the south of the island, which is protected. We carried out three 50 m linear transects along the seashore. Transects were randomly located at a minimum of 100 m from each other in order to enhance the chances of a fair sampling of most of the flora. In both areas, vegetation, and so transects, followed a clear sequence from the seashore inland. The sequence starts from therophytes in the drift line zone (Aliance *Cakilion maritimae*), very altered by the presence of tourism, followed by embryonic dune (*Agropyro‐Minuartion peploidis*) and white dune communities (*Ammophilion australis*), which lead to semi‐fixed dunes (*Crucianellion maritimae*) landwards (Llorens et al., 2021).

### 2.2 Pollinator surveys

Areas were sampled for 10 weeks, from the beginning of *E. maritimum* flowering in the first week of June until the second week of July, plus 2 weeks extra (one after and one before) in order two observe pollinator diversity variation. Surveys took place between 08:00 and 18:00 h under favorable weather conditions.

A pollinator survey involved an observer slowly walking (40 min) along a transect and recording only those insects that contacted the plant's reproductive structures while actively searching for pollen and/or nectar. As the focus was put on gathering the highest diversity of pollinators, we opted to not gather information about abundance, building qualitative (binary) networks. So, in each interaction, pollinator and plant species was noted, and photographs were taken to ensure proper identification. Search was limited to those insects belonging to the insect orders most associated with pollination (Coleoptera, Diptera, Hymenoptera and Lepidoptera). Due to the great quantity of pollinators in both areas, only first interaction per day per transect was recorded, in other to construct a presence‐absence interaction matrix per session. When floral visitors were not possible to identify they were captured and placed into individually labeled vials. To minimize our impact on local insect populations, only subsets of individuals from each non‐identified species were netted. Insects were frozen and transferred to the laboratory where they were stored until identification. Insects were identified, if possible, to species level. The observed species were compared with reference studies and with international, national and local databases (GBIF, Biodibal, BioAtles, Pollinib) to evaluate the presence of new cites and species with reduced distribution.

### 2.3 Network analysis

Sampling coverage was evaluated as an indicator of sampling completeness, using the statistical software R (R Core Team, 2013) and the package iNEXT (Hsieh et al., 2016). Three qualitative plant–pollination networks (presence and absence of interactions between taxons), were carried out, one for each population and one for the whole observations. Descriptors for structure and resilience of pollinator interactions were calculated as described by Fantinato et al. (2019) and Traveset et al. (2017) using the *bipartite* R‐based package (Dormann et al., 2008). At the network level, Connectance (*C*; Dunne et al., 2002), Nestedness (*N*; Almeida‐Neto et al., 2008), Shannon diversity Index (*S*; Shannon, 1948), Links per species or Connectivity (*LP*) and number of compartments (*NC*) were calculated. Connectance is a proportion of the observed links divided by the number of total of possible links (Dunne et al., 2002). Nestedness is measure of departure from systematic arrangement of species by niche width (Dormann et al., 2009), and is considered as the ecological tendency of specialist species to interact with a subset of species that interact with more generalist species (Almeida‐Neto et al., 2008; James et al., 2012). The Shannon diversity Index measures species diversity on the basis of species richness and evenness in abundance (Santini et al., 2017). Links per species indicates the number of different species a taxon interacts with. Finally, the number of compartments reflects the degree of clustering of the network. At the species level, Specificity (*Spec*) was considered for analysis (Poisot et al., 2012), which is considered as the coefficient of variation of interactions, and ranges from 0 (low specificity) to 1 (high specificity). Specialization level is, similarly, the level of selectiveness of a species. Using this metric, the degree of selectivity of pollinators and plant species was established (highly selective, *Spec* > 0.75; selective, 0.75 > *Spec* > 0.5; opportunistic, 0.5 > *Spec* > 0.25 and highly opportunistic, *Spec* < 0.25), as suggested by Castro‐Urgal and Traveset (2014).

For each of the three networks, total number of plant species with interactions (NP) and total number of pollinators (NS) were calculated. Same analyses were carried out after removing singletons (pollinators that visited only one species and detected once in the whole experiment), to evaluate the potential increase of specialization as a result of rare species (Blüthgen et al., 2008; Dormann et al., 2008). In order to confirm that our results described patterns that are different from random, the observed interaction network was compared with a null model based on a number of random networks (Dormann et al., 2008). To do so, 1000 null versions (null model) of each community matrix were generated using the *mgen* algorithm implemented in the *bipartite*, which returns a list of randomized matrices without keeping any variable constant (Dormann et al., 2008).

Parallelly, the diversity of pollinators per plant species per session was analyzed, in order to describe potential temporal shifts of pollinators between species.

### 2.4 Phenology

In each sampling area, plant surveys were carried out to assess the phenological distribution of plants coinhabiting with *E. maritimum*. Each sampling day a phenological survey was carried out in 10 key species of the habitat, in order to analyze the relationship between flowering and pollinator surveys. To do so, for each species individuals, inflorescences or flowers were followed and flowered units were counted as proposed in Gil (1994). For each species, a flowering peak and a standard deviation of flowering was calculated to estimate phenological curves, and then it was plotted using the ggplot2 (Wickham, 2011) package and the statistical software R.

### 2.5 Pollinators behavior

Specific observations of *E. maritimum* pollinators were carried out to assess pollinators behavior and diversity. In each transect random individuals of *E. maritimum* were selected and observations were made for 20 min each transect, with a total observation time of 20 (minutes per transects)·8 (transects per day)·4 (sampling sessions per area) = 640 min in each area. For each interaction, the number of visited capitula, its whorl and the total time spend in an individual was recorded. The number of visited capitula was evaluated across families using generalized linear models assuming a Poisson distribution, while the time spend in visits was modeled using linear models, evaluating the potential effect of species and whorls (see Cortés‐Fernández, Cerrato, Ribas‐Serra, & Gil Vives, 2022 for detailed description about whorls in the species). The effect of the above mentioned factors in response variables was evaluated using Analysis of Variance and Deviance in R (Faraway, 2002), respectively.

## 3 RESULTS

### 3.1 Pollinator surveys

**FIGURE 2:**
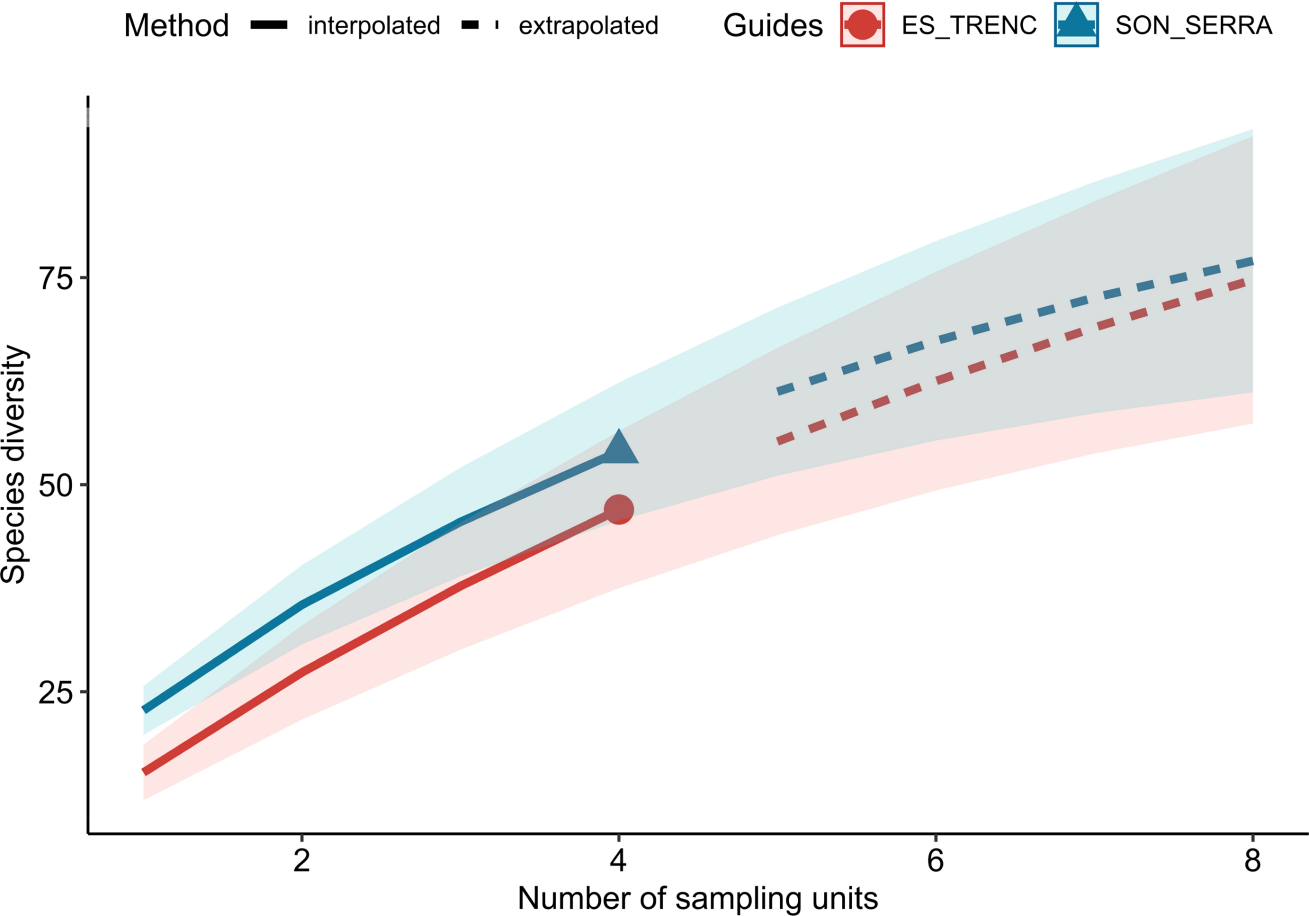
Sampling coverage per studied area. Solid lines indicate the model for the effective sampling units (sampling sessions), while dashed line indicates the prediction for future sessions.

We recorded 353 contacts, involving 71 pollinator species (Table S2) and 15 plant species. The sampling coverage showed that species richness per session was still growing, but decelerating, at the end of the sampling sessions (Figure 2). Although observed species diversity was higher in SS than in ET, nearly similar richness would be obtained in both areas after 8 sampling sessions. Diversity of pollinators and Shannon diversity Index indicated that SS was richer in pollinators than ET (Table 1), recording interactions in 15 different plant species in SS, while only 7 in ET. However, only in both populations and in SS the Shannon Index was statistically different from null models. Connectance was higher in ET than in SS, while Links per species were lower. Nestedness was higher in ET than in SS, but only significant in the matrix of both populations. The analysis of the number compartments showed that two different compartments were identified considering both populations and ET individually, while SS displayed only one compartment (but not statistically significant from null models, Table 1).

**TABLE 1:** Network metrics of the different populations (Son Serra—SS, Es Trenc—ET) and both (All) in the full network (up) and with omitted singletons (down). NS = number of pollinator species found interacting with plants, NP = number of plant species visited by potential pollinators, *C* = connectance, *S* = Shannon Diversity Index, *N* = nestedness, *LS* = links per species, *NC* = number of compartments. The significance of observed results was tested by constructing 1000 randomized networks with identical margin totals as the empirical networks and comparing the observed and random values using the null model ‘r2d’ (*p < .05).

*Population*	*NS*	*NP*	*C*	*S*	*N*	*LS*	*NC*
All	70	15	0.12*	4.88*	8.40*	1.54*	2
Es Trenc	39	7	0.19*	3.95	27.13	1.13*	2
Son Serra	48	14	0.14*	4.57*	11.83	1.56*	1
All	41	12	0.12*	4.08*	17.66	1.11*	5*
Es Trenc	19	6	0.17	2.94	29.09	0.76	6
Son Serra	25	11	0.15	3.69	26.19	1.11	4

**FIGURE 3:**
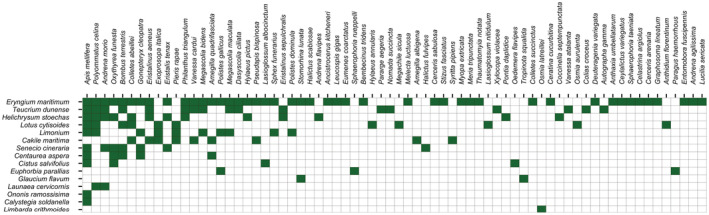
Matrix of pollinators species per plant species. Pollinators are ordered by diversity of interactions (grade of selectiveness) from left to right. In the genus matrix, color gradient indicates de diversity of taxons per interaction.

**FIGURE 4:**
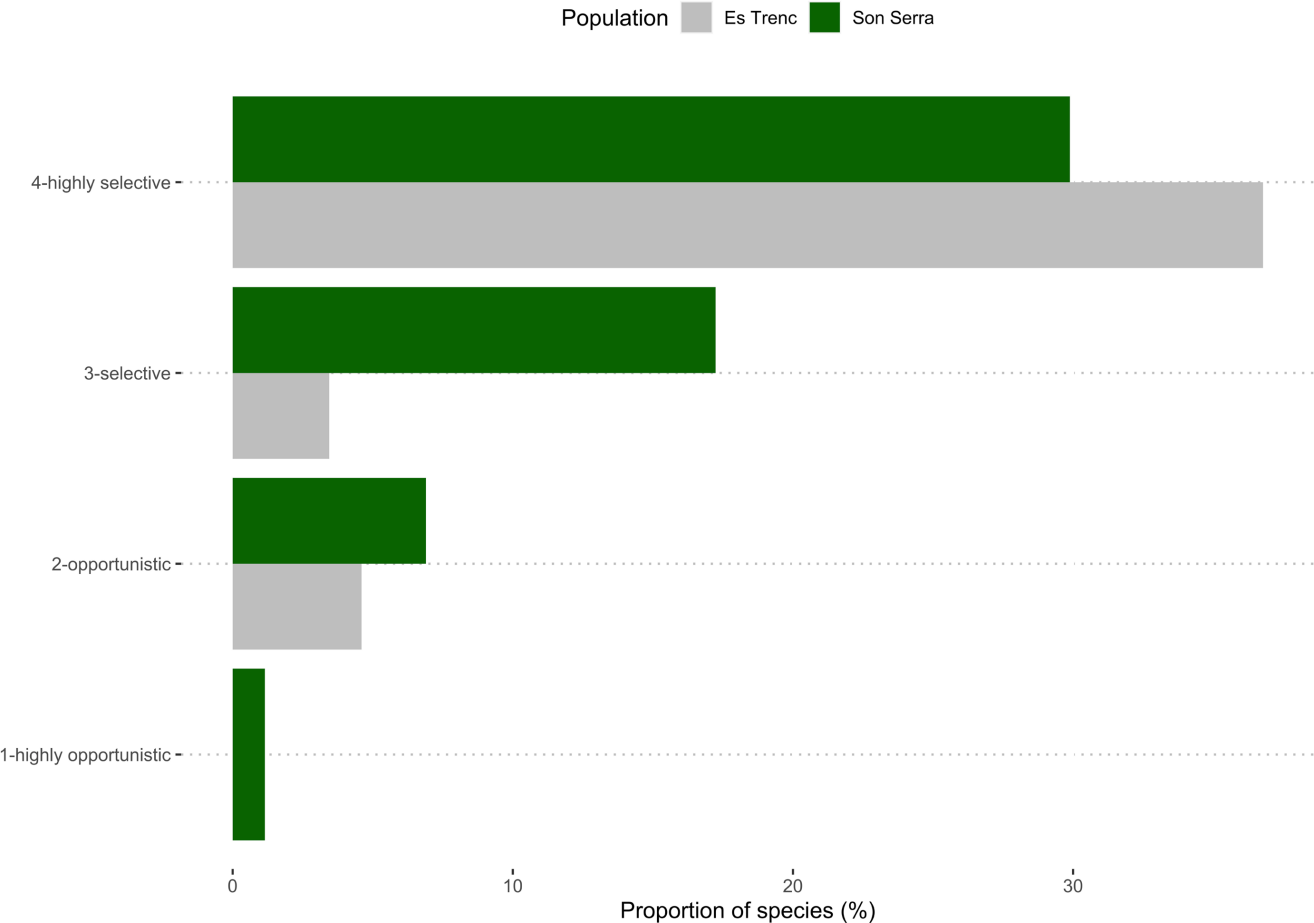
Proportions of pollinator species in the different categories of selectivity at each of the study sites. The degree of selectivity is calculated based on the *Specificity index* (highly selective, *Spec* > 0.75; selective, 0.75 > *Spec* > 0.5; opportunistic, 0.5 > *Spec* > 0.25; highly opportunistic, *Spec* < 0.25).

Network evaluation indicated that SS web was more complex than ET, displaying more nodes and links (Table 1). In both webs, *E. maritimum*, *T. dunense* and *H. stoechas* displayed the higher diversity of pollinators and interactions (Figure 3). On the other hand, in other plant species like *Ononis ramosissima* Desf., *Calystegia soldanella* (L.) R. Br. and *Limbarda crithmoides* (L.) Dumort only one pollinator species was found (Figure 3). Although no data about pollinator abundancy were gathered, less pollinators were observed in ET than in SS. On the other hand, the specificity indicated that in SS pollinators were more opportunistic than in ET (Figure 4). However, it must be considered that network from ET was not significantly different from null models, and so no clear patterns can be obtained, contrary to SS and to the full network of both areas (Table 1). Twenty‐nine species were characterized as singletons (pollinator species only observed once visiting one plant species). When omitted for the analysis, nestedness increases in SS and the number of compartments in the three networks. In this case, only with the combination of both populations variables are significantly different from null models, with the exception of Nestedness (Table 1).

*Apis mellifera* L. was the most polylectic species of pollinator, visiting 10 of the 15 plant species, while 45 pollinator species were found only visiting a single plant species (Figure 4). Of the 30 families found, *Apidae*, *Syrphidae* and *Colletidae* were the more diverse families in the network. On the other hand, two families (*Satyridae*, *Bruchinidae*) were represented by only one species. Hymenoptera was the most diverse family in the network, followed by Diptera and Lepidoptera. Three plant species presented very low levels of selectiveness, *H. stoechas*, *T. dunense* and *E. maritimum*, while *C. soldanella*, *O. ramossisima* and *L. crithmoides* presented only one interaction and were considered as highly selective (Table 2). The vast majority of pollinator species in both populations were highly selective, while high‐opportunistic species were proportionally negligible, representing <2% of species (Figure 4; Table S3). In ET highly selective and selective species were more representative than in SS, where selective and opportunistic species were more represented. Attending at pollinator families, most of them were classified as highly selective, being the only opportunistic families *Andrenidae*, *Apidae*, *Lycaenidae*, *Pieridae*, *Scarabeidae* and *Syrphidae* (Table S4; Figure S1). Considering pollinator orders, Diptera, Hymenoptera and Lepidoptera can be considered as highly opportunistic while Coleoptera can be considered as opportunistic (Table S5).

**TABLE 2:** Plant species metrics of the different populations (Son Serra, Es Trenc) And both (All). *D* = number of different pollinators species, *Spec* = Specificity. Selectiveness is calculated based on the *Specificity index* (highly selective, *Spec* > 0.75; selective, 0.75 > S*pec* >0.5; opportunistic, 0.5 > S*pec* > 0.25; highly opportunistic, *Spec* < 0.25).

Species	Es Trenc		Son Serra		All		
	*D*	*Spec*	*D*	*Spec*	*D*	*Spec*	*Specificity*
*Cakile maritima*	5	0.42	4	0.48	9	0.31	Opportunistic
*Calystegia soldanella*			1	1	1	1	Highly selective
*Centaurea aspera*			5	0.43	5	0.43	Opportunistic
*Cistus salvifolius*			4	0.48	4	0.49	Opportunistic
*Eryngium maritimum*	22	0.14	28	0.12	45	0.09	Highly opportunistic
*Euphorbia parallias*			3	0.56	3	0.57	Selective
*Glaucium flavum*			2	0.7	2	0.7	Selective
*Helichrysum stoechas*	6	0.38	11	0.27	13	0.25	Highly opportunistic
*Launaea cervicornis*			2	0.7	2	0.7	Selective
*Limbarda crithmoides*	1	1			1	1	Highly selective
*Limonium sp*.	6	0.38	5	0.43	10	0.29	Opportunistic
*Lotus cytisoides*	4	0.48	8	0.33	11	0.28	Opportunistic
*Ononis ramossisima*			1	1	1	1	Highly selective
*Senecio cineraria*			7	0.35	7	0.36	Opportunistic
*Teucrium dunense*	8	0.32	16	0.21	17	0.21	Highly opportunistic

**FIGURE 5:**
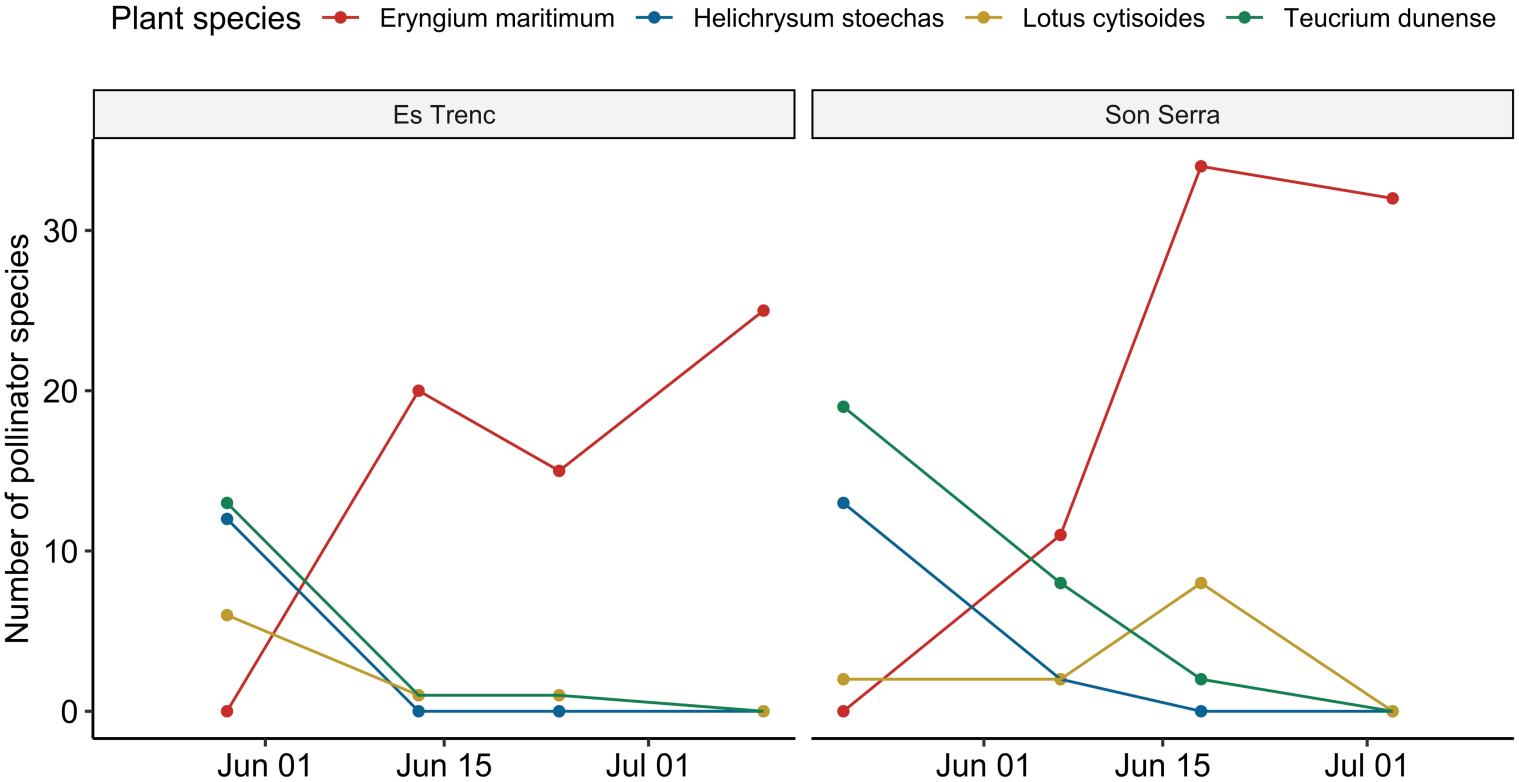
Changes in pollinator diversity of the more generalist plant species in the two studied populations. Only plant species visited by more than 10 pollinators species are indicated.

Pollination networks strongly varied among sessions (Figure 5). During first sessions, *E. maritimum* was outside of its flowering period and *T. dunense* and *H. stoechas* gathered the vast majority of interactions. Then, *T. dunense* and especially *H. stoechas* experimented a decrease while *E. maritimum* began to attract more interactions. In the third session, *E. maritimum* was already the stronger species in terms of pollinator diversity. A slight decrease in pollinator diversity was observed comparing first and last sampling sessions in each area.

### 3.2 Phenology

**FIGURE 6:**
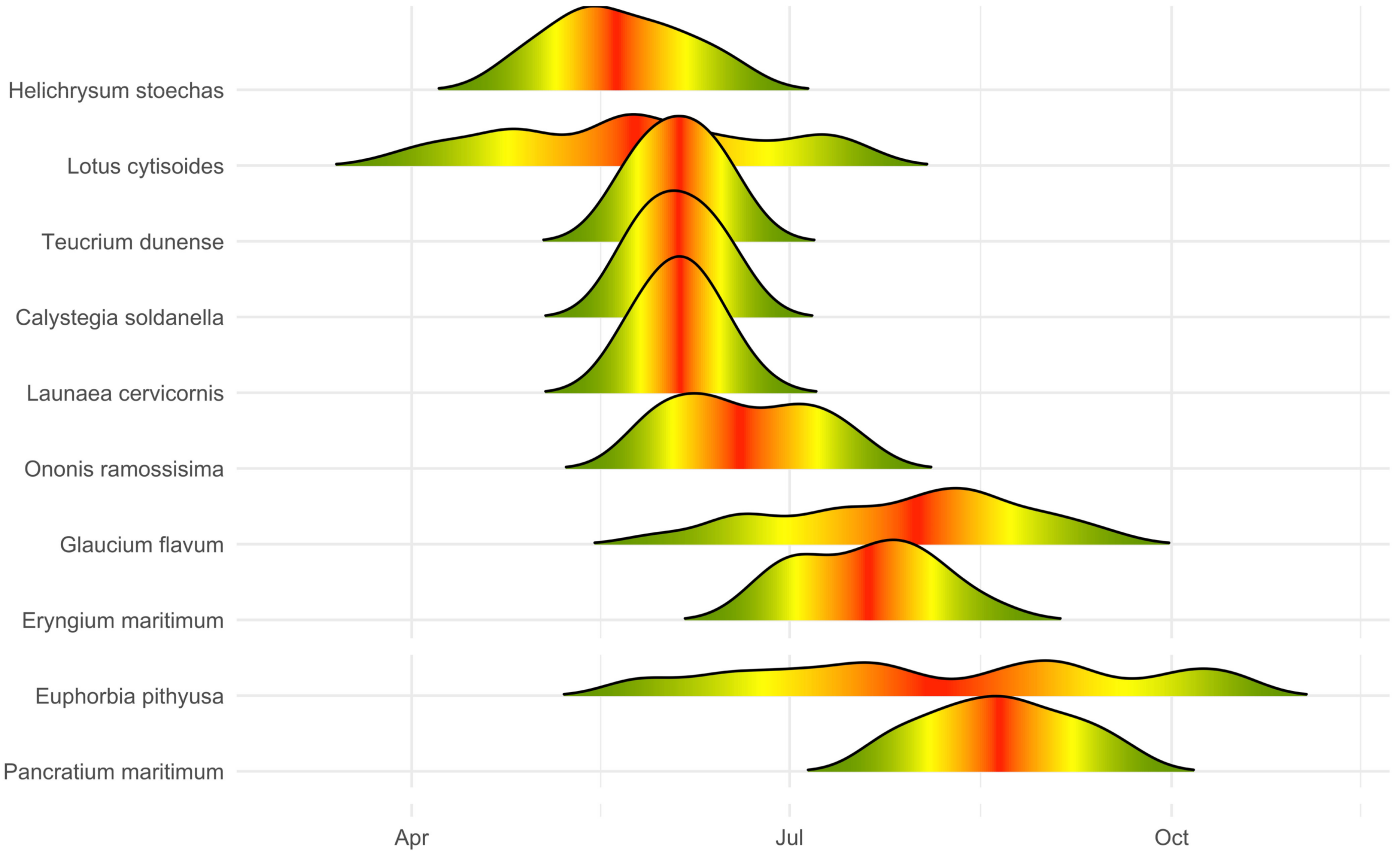
Flowering schedules of the analyzed dune species. Color gradient is used to indicate the flowering peaks. Curves indicate the number of opened flowers or flowering units in a certain time (see Gil, 1994, for specific methodological details).

Seven of the ten plant species displayed Gauss‐like phenological curves, with a defined flowering peak, while *L. cytisoides*, *G. flavum* and *E. pithyusa* presented a diffuse flowering period (Figure 6). Considering the most abundant species in the habitat, the flowering period of *E. maritimum* is located after *T. dunense* and *H. stoechas*, being the last species before *P. maritimum*. Observations of the flowering periods between SS an ET during pollinators samples, although not quantitatively evaluated, suggested that plant populations at ET flowered with a delay of, at least, 1 week respect to SS.

### 3.3 Pollination behavior

**FIGURE 7:**
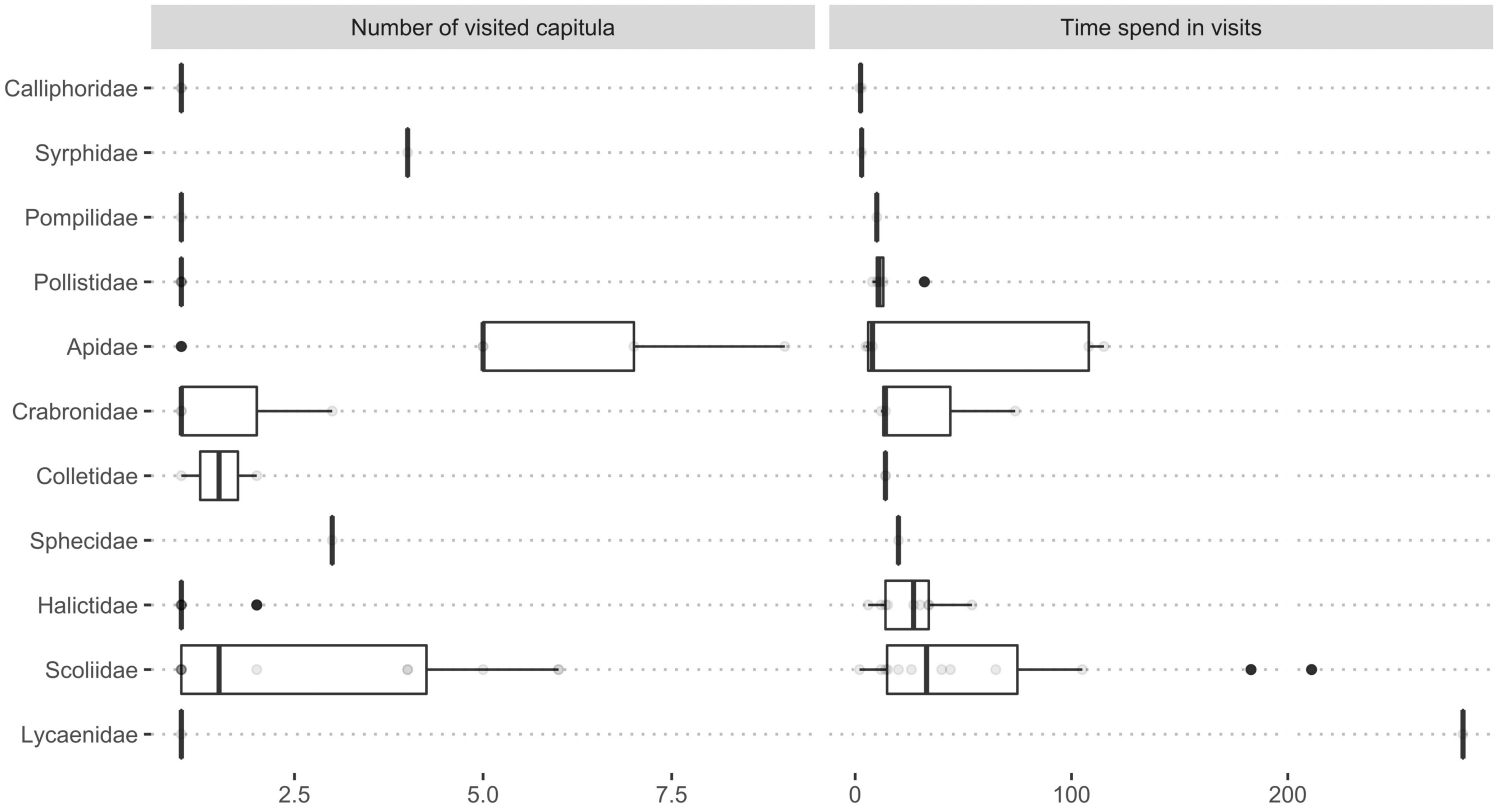
Results of the focal observation of pollinators behavior on *E. maritimum* individuals. For comparison, species have been grouped in families.

A relation between time spent in visits and the whorl of the visited capitulum was observed, decreasing the time spent in visits in outer whorls (df = 4, *F* = 2.21, *p* = .04). Time spent in visits and number of visited capitula was variable among families (Figure 7). *Apidae* pollinators visited more capitula per plant than any other family, while *Lycaenidae* pollinators spent more time per visits (Table S6). Data about some families were insufficient to analyze behavior deeper.

## 4 DISCUSSION

### 4.1 Dune pollination networks

Most plant species are generalist rather than specialists (Gómez & Zamora, 2006; Herrera, 1996), and similarly the great majority of pollinators visit a great variety of plant species (Bosch et al., 2009). The evaluation of the level of specialization is particularly important as more specialized networks are more prone to collapse (Thébault & Fontaine, 2010). Most of the species observed in the present study could be considered as specialists, as their interactions were found in one or a few plant species. However, Petanidou et al. (2008) observed than 90% of species labeled as specialist are indeed generalist when multiple‐year data are added, not being specialist but rare (less abundant). The high proportion of singletons observed is reasonable considering that the study was focused on finding the highest diversity of pollinator species possible. On the other hand, as suggested by Armbruster (2006), generalist pollinators can behave as specialist species in local scale, as a consequence of ecological specialization. Including the interactions found by pollen analysis is known to reduce the proportion of extreme specialist and increase connectance, as pollen remains in the body of pollinators for long period providing a record of visitation history rather than a single sample (Bosch et al., 2009; Courtney et al., 1982).

Two main areas, ET and SS were analyzed in order to gather the maximum diversity of pollinators and evaluate the differences between the two populations, being the first a protected area, in which users are not allowed to walk through the dune vegetation system. As a consequence of this protection character, it was expected that ET would display a more diverse network than SS, which would be more impacted by touristic pressure. However, SS presented a higher pollinator richness than ET, which could be related to differences in plant species abundance between populations, that although were not objective of the present work, were observed in the field. In this sense, floral abundance and plant diversity was higher in SS, factors that have been associated with a high density and abundance of interactions (Blüthgen et al., 2007; Hagen & Kraemer, 2010). On the other hand, a high pollinator richness can be considered as an indicator of moderate disturbance levels. As proposed by Connell (1978) in the intermediate disturbance hypothesis (IDH), moderate disturbances levels maintain the highest levels of species richness, although it should be taken into consideration with caution as IDH is considered as an oversimplification of nature (Fox, 2013). In sandy dune ecosystems, it has been documented that human disturbance increases micro‐site diversification (Slaviero et al., 2016) but, at the same time, it increases competition between pollinators and local exclusion of weaker species, as a result of a reduction of floral resources availability (Wojcik et al., 2018).

Differences in connectance between populations could be considered as an indicator of differences in populations resilience (Heleno et al., 2012), by which the protected area, ET, would be more resilient than SS. However, it is known that species richness strongly influences connectance (Fantinato et al., 2019; Olesen & Jordano, 2002). In this sense, rarefaction curves indicated that more sampling effort would have conducted similar species richness in both populations. Moreover, low values of nestedness, as occurs in SS, are associated with low levels resistance and resilience (Bastolla et al., 2009) sometimes as a result of intense disturbances (Revilla et al., 2015; Welti & Joern, 2018), although not always (Spiesman & Inouye, 2013). However, when singletons are omitted, SS displays similar, but slightly lower, nestedness values than ET, but cannot be further discussed as they are not statistically significant from null models. Considering these findings, no clear effect of protection measures on pollination networks can be extracted from our results, and so they should be evaluated in future studies.

Hymenoptera was the most diverse order of pollinators in the dunes, which is consistent with the observations of previous studies (Castro‐Urgal & Traveset, 2014; Fantinato, Del Vecchio, Silan, & Buffa, 2018). Other orders, such as Diptera and Lepidoptera, also played an important role in dune pollination (Gil, 1994). In our study areas, Apidae were the most diverse family of pollinators and the most opportunistic. This is rational considering than bees are generally polylectic, usually alternating visits between a pollen‐rich source and a nectar‐rich source (Bosch et al., 2009) and exploiting pollen resources from various species (Minckley & Roulston, 2006), although even oligolectic bees visit various species for nectar.

An invasive plant species, *Senecio cineraria* DC., was found in our sampling sessions in SS. Attending to the observed interactions, it behaves as a generalist species, although one pollinator species, *Halictus fulvipes*, was only found exploiting its floral resources. Previous studies carried out in a very close area suggested that alien species (in this case *Carpobrotus edulis* (L.) L. Bolus) influence the quantitative component of pollination, influencing negatively (competition) or positively (facilitation; Moragues & Traveset, 2005). So, the present results describe the effect of another alien species, quite abundant in the Balearic Island coasts due to its commercialization as garden plant, producing pollinator species displacements from autochthonous to allochthonous species, altering the structure of coastal networks.

### 4.2 Phenology

A strong phenological variation was present among sampling sessions. For most pollinator species, flowering phenology is the main driver for pollinator distribution rather than flower traits (Bosch et al., 1997), usually presenting short activity periods of pollination (Farré‐Armengol et al., 2015). However, a few species presented irregular and long phenological periods (*L. cytisoides*, *G. flavum* and *E. pithysusa*). In this sense, bivoltine insects have been proposed to be especially dependent upon plants with long or late flowering periods, as in *E. maritimum* (Howe et al., 2010). Although *L. cytisoides* and *E. pithyusa* are strongly entomogamous, *G. flavum* presents higher levels of autocompatibility (Gil, 1994), which could be related with their irregular phenology and the low pollinator diversity observed in the species, which is particularly visited by coleoptera.

The phenological distribution of species with very low selectiveness (*H. stoechas*, *T. dunense* and *E. maritimum*) suggests that there is a selective pressure to decouple flowering between species as to avoid interspecific competition for pollinators. At community level, competition is thought to be the primary selective force molding flowering schedules (Rathcke, 2014; Waser, 1978). However, when pollinator abundance is optimal, also facilitation among species can occur (Rathcke, 1983), as a sequential mutualism, in which early‐flowering species support pollinators of late‐flowering species (Waser & Real, 1979), or as a result of synchronous blooms that attract more pollinators that single species alone (Rathcke, 1983). Differences in phenological timing between populations could be related with temperature, as southern coastal populations due to sea currents are colder (Guijarro, 1986; Table S7), which is known to produce a delay in flowering (Gil, 1994; Llorens et al., 2021). On the other hand, the effect of wind is known to have a great impact in pollinators, which enhances the importance of multiannual data to reduce the potential impact of this variable. On the other hand, more focus should be put on pollinator species abundance in both areas, because attending to our observations strongly varied among sampling sessions.

### 4.3 Pollinator behavior

Remarkable differences were found between families attending at pollination behavior. Apidae, the most represented family in both populations, visited more capitula per foraging bout, spending few time per visit, which is consistent with the results of previous studies (Brunet, 2009). The high variability observed in the behavior of Apidae could be attributed to the presence of different functional groups within the family (i.e., bumble‐bees, solitary bees and social bees) with usually present different pollination behaviors, spending bumble‐bees less time per visit but interacting with more flowers per visit (Brunet, 2009). The high values of time per visit observed in Lepidoptera, overall in *Polyommatus celina*, could be attributed to behavioral thermoregulation (Kevan & Shorthouse, 1970), and not really to extensive periods of foraging. In this sense, it must be considered that not all insect visitors may actually be pollinators, and also that pollination is not equally probable among pollinator species, due to differences in carrying capacity, morphology, foraging behavior and the degree of fidelity (Lindsey, 1984). For example, Coleoptera is known to visit a great variety of plant species, but at the same time is generally considered to have a low effective pollination rate (Sayers et al., 2019; Thayer et al., 2003). However, the evaluation of all these parameters in a single study is prohibitive, and so simplifications, as we did, should be carried out.

### 4.4 The pollinators of *E. maritimum*

As suggested by our previous observations, *E. maritimum* is strongly entomophilous (Table 3; Cortés‐Fernández, Cerrato, Ribas‐Serra, & Gil Vives, 2022), being visited by a wide variety of pollinators. Forty‐five species of 29 families can be considered as potential pollinators of the species, being Diptera and Hymenoptera the most diverse orders. Although some of the detected species have only a few cites in GBIF (Figure S2), all the detected plant and animal species in the study were previously cited Mallorca, except for *Leucospis gigas* and *Odice blandula* (Table S2; Figure S3). *Leucospis gigas* is an hymenoptera of the family Leucospidae widely distributed in the warmer parts of the Paleartic Region, as proposed by Madl and Schwarz (2014). In this same study, they propose that *Leucospis gigas* is found in the Balearic Islands, but no exact location or island is provided. So, to our knowledge, it is the first cite of the species in Mallorca, which was found only once in SS pollinating *E. maritimum*. On the other hand, *Odice blandula*, is a Lepidoptera of the family Erebidae which was detected previously in Ibiza and Formentera (Férriz et al., 2006), but similarly, no references about its presence in Mallorca are available, although its distribution in the island is known within the local experts (Truyols, pers. commun.)

**TABLE 3:** Pollinator species detected in *Eryngium maritimum*. Reference indicates if the pollinator species was previously detected in the literature as a potential pollinator. Hegi (1935) [1], Gil (1994) [2], Cortés‐Fernández, Cerrato, Ribas‐Serra, and Gil Vives (2022) [3] and Polinib Database [4]

Order	Family	Genus	Species	ET	SS	References
*Coleoptera*	*Buprestidae*	*Anthaxia*	*Anthaxia umbellatarum*	1	0	[3]
	*Pentatomidae*	*Graphosoma*	*Graphosoma lineatum*	1	0	
	*Scarabaeidae*	*Oxythyrea*	*Oxythyrea funesta*	0	1	
*Diptera*	*Calliphoridae*	*Lucilia*	*Lucilia sericata*	1	0	
		*Stomorhina*	*Stomorhina lunata*	0	1	
	*Cloropidae*	*Thaumatomyia*	*Thaumatomyia notata*	1	0	
		*Myopa*	*Myopa extricata*	0	1	
	*Pompilidae*	*Deuteragenia*	*Deuteragenia variegata*	0	1	
	*Syrphidae*	*Eristalinus*	*Eristalinus aeneus*	0	1	
		*Eristalis*	*Eristalis tenax*	0	1	
		*Sphaerophoria*	*Sphaerophoria taeniata*	1	0	
*Hymenoptera*	*Andrenidae*	*Andrena*	*Andrena agilissima*	0	1	
			*Andrena morio*	1	1	
	*Apidae*	*Amegilla*	*Amegilla quadrifasciata*	0	1	
		*Apis*	*Apis mellifera*	0	1	[3–4]
		*Bombus*	*Bombus terrestris*	0	1	[3–4]
		*Ceratina*	*Ceratina cucurbitina*	1	0	
		*Melecta*	*Melecta luctuosa*	0	1	
	*Colletidae*	*Colletes*	*Colletes abeillei*	0	1	[2]
			*Colletes succinctus*	1	0	
		*Hylaeus*	*Hylaeus pictus*	1	0	
	*Crabronidae*	*Cerceris*	*Cerceris arenaria*	1	0	[1]
			*Cerceris sabulosa*	1	0	
		*Philanthus*	*Philanthus triangulum*	0	1	[2–3‐4]
		*Stizus*	*Stizus fasciatus*	0	1	
	*Halictidae*	*Ceylalictus*	*Ceylalictus variegatus*	1	0	[4]
		*Halictus*	*Halictus scabiosae*	1	1	[3]
		*Lasioglossum*	*Lasioglossum albocinctum*	0	1	
		*Pseudapis*	*Pseudapis bispinosa*	1	0	[4]
	*Leucospidae*	*Leucospis*	*Leucospis gigas*	0	1	
	*Polistidae*	*Bembecinus*	*Bembecinus tridens*	0	1	
		*Polistes*	*Polistes dominula*	1	1	[4]
			*Polistes gallicus*	1	0	[2–4]
	*Pompilidae*	*Entomobora*	*Entomobora fuscipennis*	1	0	
	*Scoliidae*	*Dasyscolia*	*Dasyscolia ciliata*	0	1	
		*Megascolia*	*Megascolia bidens*	1	1	
			*Megascolia maculata*	1	1	[3–4]
	*Sphecidae*	*Sphex*	*Sphex funerarius*	0	1	
	*Typhiidae*	*Meria*	*Meria tripunctata*	1	0	[3]
	*Vespidae*	*Ancistrocerus*	*Ancistrocerus kitcheneri*	0	1	
	*Vespidae*	*Eumenes*	*Eumenes coarctatus*	1	0	
*Lepidoptera*	*Lycaenidae*	*Celastrina*	*Celastrina argiolus*	0	1	[3]
		*Polyommatus*	*Polyommatus celina*	1	0	[4]
	*Nymphalidae*	*Vanessa*	*Vanessa cardui*	0	1	[3]
	*Pieridae*	*Gonepteryx*	*Gonepteryx cleopatra*	0	1	

	*Lycaenidae*	*Polyommatus*	*Polyommatus celina*	281	1
	*Nymphalidae*	*Vanessa*	*Vanessa cardui*	79	

*Eryngium maritimum* can be considered as an extreme opportunistic species, which is consistent with previous observations carried out in northern populations (Fitter & Peat, 1994; Hegi, 1935; Westrich, 2001; Zanella et al., 2009). Apiaceae species are known to be visited by a large quantity of insects (Davila, 2006; Zych et al., 2019) as a result of not presenting floral restrictions accessing to pollen and nectar (Lindsey, 1984). While myophile pollination is known to be usually focused on species with little odorless flowers, psychophyle pollination is carried out preferentially in species with more intense aromas and big tubular flowers (Aguado Martín et al., 2015). In our study, both orders exploited floral resources of a high diversity of plant species, including *E. maritimum*, but attending to flower morphology the species would not be optimal for lepidoptera. In previous quantitative studies carried out in other *Eryngium* and Apiaceae species, flies, bees and beetles made up the majority of insect visitors while butterflies and moths were rarely observed (Danderson & Molano‐Flores, 2010; Zych, 2007), which is consistent with our results and field observations, but should be proved in future studies considering specifical pollinator abundance.

The species presented the greater pollinator richness in both populations. This is logical considering that the focus of the study was put on the species, and as a consequence of oversampling, the diversity of *E. maritimum* pollinators in relation with the other species could be overestimated, as the study comprises its whole flowering period. However, a central role of the species can be defined considering the diversity of pollinators that exploit the floral resources of the species, in a period in which *E. maritimum* is the last generalist species in flower previously to the arrival of cold temperatures. In this sense, *P. maritimum*, which flowers after *E. maritimum*, is not functionally similar, as it is known to be strongly related to evening and nocturnal rather than diurnal pollinators (Eisikowitch & Galil, 1971). So, it can be concluded that conservation of *E. maritimum* is important in terms of dune pollinators conservation, as a vast variety of pollinators depend on the species at the end of the flowering season, previously to the decrease in activity due to temperature changes (Mellanby, 1939; Taylor, 1963). However, the specific functionality of the species in the dune pollination network should be evaluated incorporating abundance data, as our study have already focused on diversity. Finally, our results suggest that it is improbable that the decrease in fruit and seed set in northern European populations could be attributed to specific pollinator extinctions, as the species behaves as an extremely opportunistic in terms of pollination. However, as suggested by Armbruster (2006), some species can behave as a specialist locally, so replicas of this study in northern populations would be critical to evaluate a potential cause of its decrease in fitness.

## 5 CONCLUSIONS

1. *Eryngium maritimum* is a widely generalist species, mainly pollinated by Diptera and Hymenoptera.
2. It flowers after the other two main generalist species, *H. stoechas* and *T. dunense*.
3. The pollination network of the protected area (ET) did not present clear differences with the non‐protected area (SS), displaying a lower diversity but higher connectance values.
4. Hymenoptera are the main pollinators of dunes, although Diptera and Lepidoptera also play a major role.
5. *Eryngium maritimum* plays an important role as it is the last generalist species in flower before the end of the dune flowering season.

## AUTHOR CONTRIBUTIONS

**Arnau Ribas‐Serra:** Conceptualization (equal); investigation (equal); supervision (equal); validation (equal); writing – review and editing (equal). **Xavier Canyelles Ferrà:** Conceptualization (equal); investigation (equal); methodology (equal); validation (lead). **Iván Cortés‐Fernández:** Conceptualization (equal); data curation (lead); formal analysis (lead); investigation (lead); methodology (equal); project administration (equal). **Lorenzo Gil:** Conceptualization (equal); funding acquisition (lead); project administration (equal); supervision (lead); writing – review and editing (lead). **Marcello Dante Cerrato:** Conceptualization (equal); investigation (equal); methodology (equal); resources (equal); validation (equal); writing – review and editing (equal).

## CONFLICT OF INTEREST

The authors declare that they have no known competing financial interests or personal relationships that could have appeared to influence the work reported in this article.

## Supporting information


Figure S1
Figure S2.
Figure S3
Table S1
Table S2
Table S3
Table S4
Table S5
Table S6

## Data Availability

Data are available from the Dryad Digital Repository https://doi.org/10.5061/dryad.p8cz8w9s8.
