# Supplementary material for: The role of E. maritimum (L.) in the dune pollination network of the Balearic Islands
Source: Ecol Evol. 2022 Aug 4;12(8):e9164. doi: 10.1002/ece3.9164 (PMC9353020; doi:10.1002/ece3.9164)
Supplement: Supplementary file 1 — Figure S1 Figure S2. Figure S3 Table S1 Table S2 Table S3 Table S4 Table S5 Table S6 [file ECE3-12-e9164-s001.docx]

SUPPLEMENTARY FIGURES


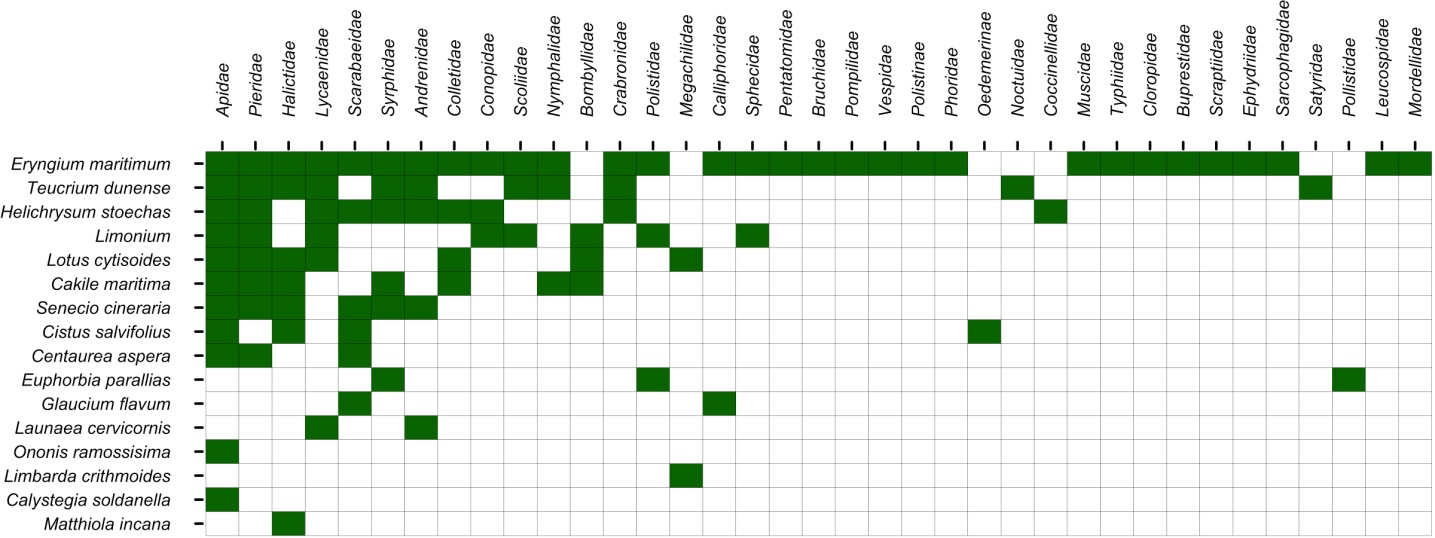


**Figure S1**. Matrix of pollinators families per plant species. Pollinators are ordered by diversity of interactions (grade of selectiveness) from left to right. The colour gradient indicates de diversity of taxons per interaction.


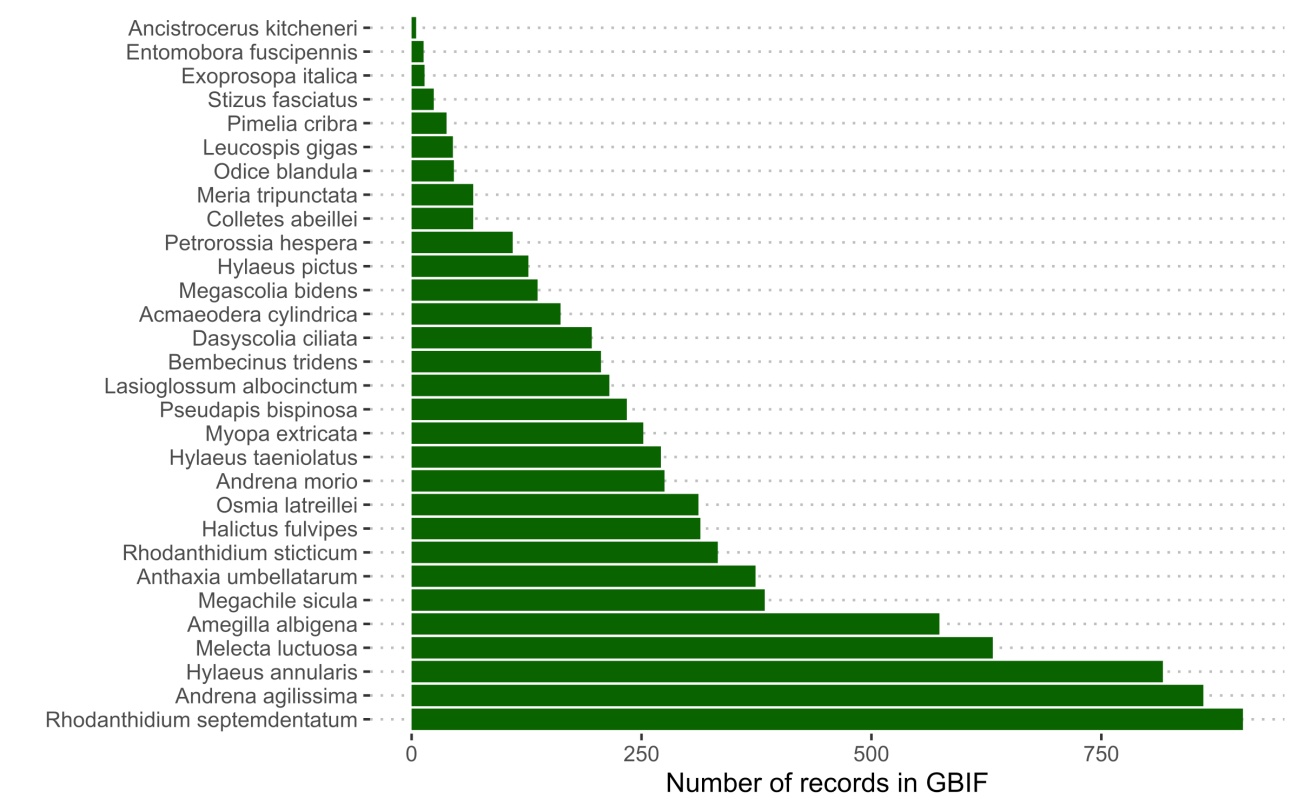


**Figure S2.** Pollinator species detected in the study with less occurrences in GBIF.


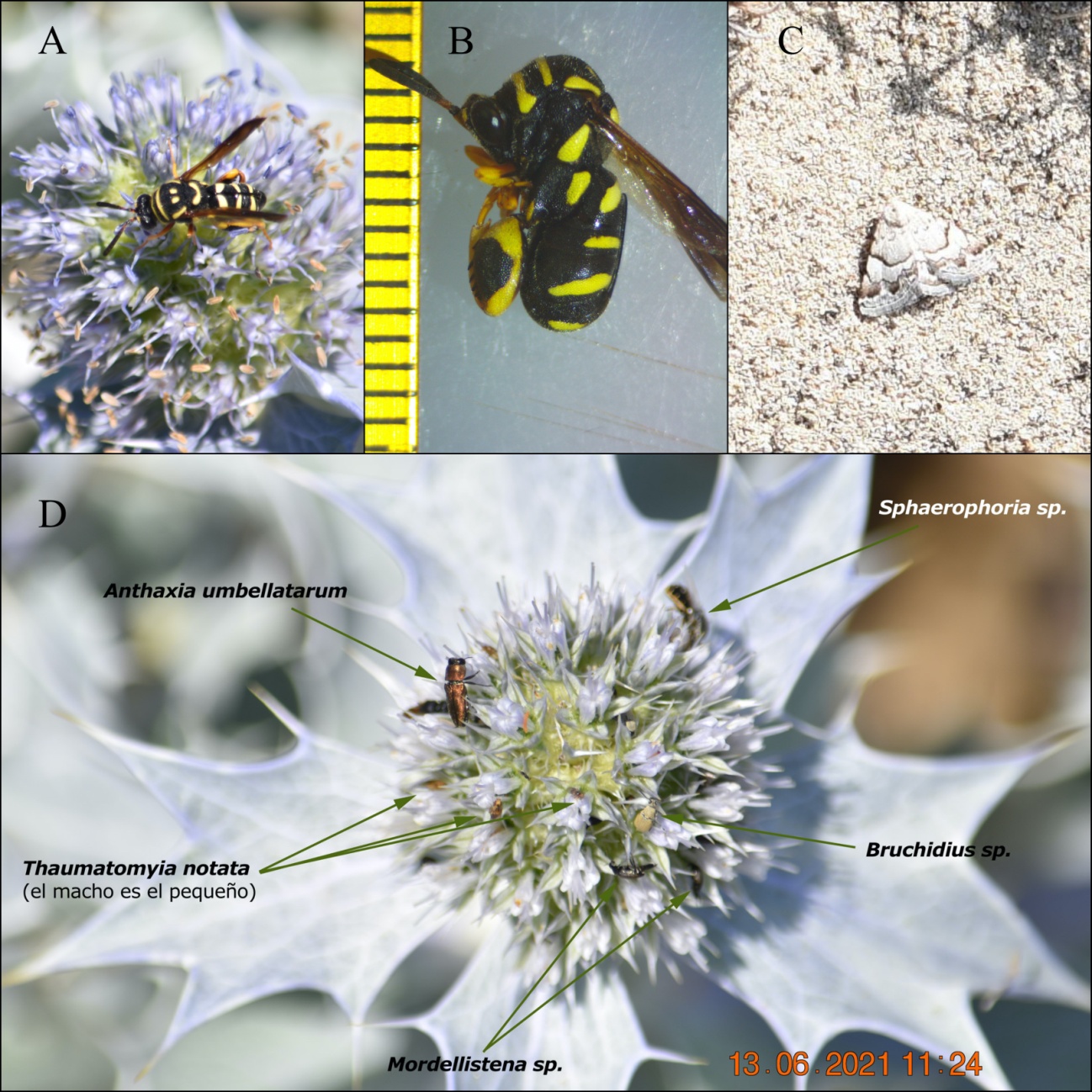


**Figure S3.** *Leucopsis gigas* pollinating *Eryngium maritimum* (A) and body detail (B). Individual of *Odice blandula* laying in the ground (C). Photography of multiple pollinator species on *E. maritimum*. Date, hour and species are indicated (D).

SUPPLEMENTARY FILES

**Table S1**. Climatic data of SS and ET populations. Data collection has been restricted until the end of the fruiting season in final August of 2021. This data complements the analysis of Guijarro (1986), which suggested that southern dunes are colder than northern Mallorcan dunes. Data source: Wunderground (Code: ISANTA224) and Balearsmeteo (Club Nautico del Sa Rapita de Campos), respectively.

|  | ES TRENC (ET) | | | | | |
| --- | --- | --- | --- | --- | --- | --- |
|  | Mean T (ºC) | Absolute min. T  (ºC) | Mean min. T  (ºC) | Absolute max. T  (ºC) | Mean max. T  (ºC) | Precipitation (mm) |
| January | 10,8 | -1,4 | 6,3 | 19,4 | 14,5 | 29 |
| February | 13,1 | 2,9 | 8,1 | 21,5 | 16,8 | 2 |
| March | 12,7 | 3,6 | 8 | 21,5 | 16,8 | 47,2 |
| April | 14,5 | 1,9 | 10,3 | 21,9 | 18,1 | 8,6 |
| May | 18,1 | 10,6 | 13,9 | 27,1 | 21,4 | 65,2 |
| June | 23,4 | 14,7 | 18,2 | 35 | 27,5 | 35 |
| July | 25,3 | 17,3 | 20,6 | 33,1 | 29 | 0 |
| August | 26,3 | 18,8 | 21,9 | 37,8 | 30,6 | 10 |

|  | SON SERRA DE MARINA (SS) | | | | | |  |
| --- | --- | --- | --- | --- | --- | --- | --- |
|  | Mean T (ºC) | Absolute min. T  (ºC) | Mean  min. T  (ºC) | Absolute max. T  (ºC) | Mean max. T  (ºC) | Precipiation (mm) | |
| January | 10,4 | -0,1 | 5,8 | 25,3 | 15,6 | 66,97 | |
| February | 12,8 | 2,9 | 7,8 | 25,1 | 18,2 | 3,6 | |
| March | 12,8 | 4,5 | 7,5 | 23,8 | 17,5 | 40,6 | |
| April | 13,8 | 2 | 9,6 | 23,5 | 19 | 8,9 | |
| May | 17,2 | 15 | 13,6 | 27,9 | 22,8 | 51,6 | |
| June | 23,8 | 15 | 17,3 | 34 | 28,1 | 46,7 | |
| July | 26 | 16,6 | 20,4 | 38,1 | 32,1 | 8,7 | |
| August | 26,1 | 18 | 21,6 | 40,8 | 31,5 | 60,9 | |

**Table S2**. List of the pollinator taxons observed per population. Ones and zeroes denote presence and absence of interactions. Endemic species are indicated with an asterisk *. In references, previous cites of species on GBIF [https://www.gbif.org , 1], Baldock et. al (2020) [2], Vives (1994) [3], Tolrà (2002)[4], Cobos [5], Gomila (1999) [6], Ebejer (2006) [7], Polinib Database [http://polinib.info/?lang=en, 8], Biodibal [https://biodibal.uib.cat/ca/, 9] and Bioatles [http://bioatles.caib.es, 10] are indicated.

| *Order* | *Family* | *Genus* | *Species* | *ET* | *SS* | *References* |
| --- | --- | --- | --- | --- | --- | --- |
| *Coleoptera* |  |  |  | 1 | 1 |  |
|  | *Bruchidae* |  |  | 1 | 0 |  |
|  |  | *Bruchidius* |  | 1 | 0 |  |
|  | *Buprestidae* |  |  | 1 | 0 |  |
|  |  | *Acmaeodera* |  | 1 | 0 |  |
|  |  |  | *Acmaeodera cylindrica*  *(Fabricius, 1775)* | 1 | 0 | [1-5-8-9-10] |
|  |  | *Anthaxia* |  | 1 | 0 |  |
|  |  |  | *Anthaxia umbellatarum* (Fabrizius, 1787) | 1 | 0 | [5] |
|  | Coccinellidae |  |  | 1 | 1 |  |
|  |  | Coccinella |  | 1 | 1 |  |
|  |  |  | *Coccinella septempunctata* L. (1758) | 1 | 1 | [1-8-9-10] |
|  |  | *Exochomus* |  | 1 | 0 |  |
|  | *Mordellidae* |  |  | 1 | 0 |  |
|  |  | *Mordellistena* |  | 1 | 0 |  |
|  | *Oedemerinae* |  |  | 0 | 1 |  |
|  |  | *Oedemera* |  | 0 | 1 |  |
|  |  |  | *Oedemera flavipes* (Fabricius, 1758) | 0 | 1 | [1-8-10] |
|  | *Pentatomidae* |  |  | 1 | 0 |  |
|  |  | *Graphosoma* |  | 1 | 0 |  |
|  |  |  | *Graphosoma lineatum*  (Müller, 1766) | 1 | 0 | [1-8-10] |
|  | *Scarabaeidae* |  |  | 0 | 1 |  |
|  |  | *Oxythyrea* |  | 0 | 1 |  |
|  |  |  | *Oxythyrea funesta*  (Poda, 1761) | 0 | 1 | [1-8-9-10] |
|  |  | *Tropinota* |  | 0 | 1 |  |
|  |  |  | *Tropinota squalida*  (Scopoli, 1783) | 0 | 1 | [1-8-9-10] |
|  | *Scraptiidae* |  |  | 1 | 0 |  |
|  |  | *Anaspis* |  | 1 | 0 |  |
|  | *Tenebrionidae* |  |  | 0 | 1 |  |
|  |  | *Pimelia* |  | 0 | 1 |  |
|  |  |  | *Pimelia*  *cribra**  (Solier, 1836) | 0 | 1 | [1-6-9-10] |
| *Diptera* |  |  |  | 1 | 1 |  |
|  | *Asilidae* |  |  | 1 | 0 |  |
|  |  | *Machimus* |  | 1 | 0 |  |
|  | *Bombyllidae* |  |  | 1 | 1 |  |
|  |  | *Exoprosopa* |  | 1 | 1 |  |
|  |  |  | *Exoprosopa italica*  (Rossi, 1794) | 1 | 1 | [1-4] |
|  |  | *Petrorossia* |  | 1 | 0 |  |
|  |  |  | *Petrorossia hespera*  (Rossi, 1790) | 1 | 0 | [1-4-8] |
|  | *Calliphoridae* |  |  | 1 | 1 |  |
|  |  | *Lucilia* |  | 1 | 0 |  |
|  |  |  | *Lucilia*  *sericata*  (Meigen, 1826) | 1 | 0 | [1-4-8] |
|  |  |  | *Lucilia*  *silvarum*  (Meigen, 1826) | 1 | 0 | [1-8-10] |
|  |  | *Stomorhina* |  | 1 | 1 |  |
|  |  |  | *Stomorhina lunata*  (Fabricius, 1805) | 1 | 1 | [1-4-8] |
|  | *Cloropidae* |  |  | 1 | 0 |  |
|  |  | *Thaumatomyia* |  | 1 | 0 |  |
|  |  |  | *Thaumatomyia notata*  (Meigen, 1830) | 1 | 0 | [7-10] |
|  | *Conopidae* |  |  | 0 | 1 |  |
|  |  | *Myopa* |  | 0 | 1 |  |
|  |  |  | *Myopa*  *extricata*  Collin, 1960 | 0 | 1 | 4 |
|  | *Ephydriidae* |  |  | 1 | 0 |  |
|  | *Muscidae* |  |  | 0 | 1 |  |
|  | *Phoridae* |  |  | 1 | 0 |  |
|  |  | *Megaselia* |  | 1 | 0 |  |
|  | *Pompilidae* |  |  | 1 | 1 |  |
|  |  | *Deuteragenia* |  | 0 | 1 |  |
|  |  |  | *Deuteragenia variegata  (Linnaeus, 1758)* | 0 | 1 | [1] |
|  | *Sarcophagidae* |  |  | 1 | 1 |  |
|  |  | *Sarcophaga* |  | 1 | 1 |  |
|  | *Syrphidae* |  |  | 1 | 1 |  |
|  |  | *Eristalinus* |  | 1 | 1 |  |
|  |  |  | *Eristalinus aeneus*  (Scopoli, 1763) | 1 | 1 | [1-4-8-10] |
|  |  |  | *Eristalinus sepulchralis*  (Linnaeus, 1758) | 0 | 1 | [1-4-8-10] |
|  |  |  | *Eristalinus taeniops*  (Wiedemann, 1818) | 0 | 1 | [4-8-10] |
|  |  | *Eristalis* |  | 1 | 1 |  |
|  |  |  | *Eristalis*  *tenax*  (Linnaeus, 1758) | 0 | 1 | [1-4-8-10] |
|  |  | *Eumerus* |  | 0 | 1 |  |
|  |  | *Paragus* |  | 0 | 1 |  |
|  |  |  | *Paragus haemorrhous*  Meigen, 1822 | 0 | 1 | [4-10] |
|  |  | *Sphaerophoria* |  | 1 | 1 |  |
|  |  |  | *Sphaerophoria rueppelli*  (Wiedemann, 1830) | 0 | 1 | [1-4-8-10] |
|  |  |  | Sphaerophoria *taeniata*  (Linnaeus, 1758) | 1 | 0 | [1-4] |
|  |  | *Syritta* |  | 1 | 0 |  |
|  |  |  | *Syritta*  *pipiens*  (Linnaeus, 1758) | 1 | 0 | [1-4-8-10] |
| *Hymenoptera* |  |  |  | 1 | 1 |  |
|  | *Andrenidae* |  |  | 1 | 1 |  |
|  |  | *Andrena* |  | 1 | 1 |  |
|  |  |  | *Andrena agilissima*  (Scopoli, 1770) | 0 | 1 | [1-2-8-10] |
|  |  |  | *Andrena*  *flavipes*  Panzer, 1799 | 1 | 0 | [1-2-8-10] |
|  |  |  | *Andrena*  *morio*  Brullé, 1832 | 1 | 1 | [1-2-8-10] |
|  | *Apidae* |  |  | 1 | 1 |  |
|  |  | *Amegilla* |  | 1 | 1 |  |
|  |  |  | *Amegilla albigena*  (Lepeletier, 1841) | 1 | 0 | [1-10] |
|  |  |  | *Amegilla quadrifasciata*  (de Villers, 1789) | 0 | 1 | [1-2-8-10] |
|  |  | *Anthophora* |  | 0 | 1 |  |
|  |  | *Apis* |  | 1 | 1 |  |
|  |  |  | *Apis*  *mellifera*  Linnaeus, 1758 | 1 | 1 | [1-2-8-9-10] |
|  |  | *Bombus* |  | 0 | 1 |  |
|  |  |  | *Bombus terrestris*  Linnaeus, 1758 | 0 | 1 | [1-2-8-9-10] |
|  |  | *Ceratina* |  | 1 | 0 |  |
|  |  |  | *Ceratina cucurbitina*  (Rossi, 1792) | 1 | 0 | [1-2-8-10] |
|  |  | *Megachile* |  | 0 | 1 |  |
|  |  | *Melecta* |  | 0 | 1 |  |
|  |  |  | *Melecta*  *luctuosa*  (Scopoli, 1770) | 0 | 1 | [2-9-10] |
|  |  | *Nomada* |  | 1 | 0 |  |
|  |  |  | *Nomada succincta*  Panzer, 1798 | 1 | 0 | [1-2-8] |
|  |  |  |  |  |  |  |
|  |  |  |  |  |  |  |
|  |  | *Thyreus* |  | 0 | 1 |  |
|  |  | *Xylocopa* |  | 0 | 1 |  |
|  |  |  | *Xylocopa violacea*  (Linnaeus, 1758) | 0 | 1 | [1-2-8-9-10] |
|  | *Crabronidae* |  |  | 1 | 1 |  |
|  |  | *Cerceris* |  | 1 | 0 |  |
|  |  |  | *Cerceris*  *arenaria*  (Linnaeus, 1758) | 1 | 0 | [1-2-8-10] |
|  |  |  | *Cerceris sabulosa*  (Panzer, 1799) | 1 | 0 | [1-2-8-10] |
|  |  | *Oxybelus* |  | 1 | 0 |  |
|  |  | *Philanthus* |  | 1 | 1 |  |
|  |  |  | *Philanthus triangulum*  (Fabricius, 1775) | 1 | 1 | [1-2-8-10] |
|  |  | *Stizus* |  | 0 | 1 |  |
|  |  |  | *Stizus*  *fasciatus*  (Fabricius, 1781) | 0 | 1 | [1-2-10] |
|  |  | *Tachysphex* |  | 1 | 1 |  |
|  | *Colletidae* |  |  | 1 | 1 |  |
|  |  | *Colletes* |  | 1 | 1 |  |
|  |  |  | *Colletes*  *abeillei*  (Pérez, 1903) | 1 | 1 | [1-2-8-10] |
|  |  |  | *Colletes succinctus*  (Linnaeus, 1758) | 1 | 0 | [1-2-8-10] |
|  |  | *Hylaeus* |  | 1 | 1 |  |
|  |  |  | *Hylaeus annularis*  (Kirby, 1802) | 0 | 1 | [1-2-8-10] |
|  |  |  | *Hylaeus*  *pictus*  (Smith, 1853) | 1 | 1 | [1-2-8-10] |
|  |  |  | *Hylaeus punctatus*  (Brullé, 1832) | 1 | 0 | [1-2-8-10] |
|  |  |  | *Hylaeus taeniolatus*  (Förster, 1871) | 1 | 0 | [1-2-8-10] |
|  | *Halictidae* |  |  | 1 | 1 |  |
|  |  | *Ceylalictus* |  | 1 | 0 |  |
|  |  |  | *Ceylalictus variegatus*  (Olivier, 1789) | 1 | 0 | [1-2-8-10] |
|  |  | *Halictus* |  | 1 | 1 |  |
|  |  |  | *Halictus*  *Fulvipes*  (Klug, 1817) | 0 | 1 | [1-2-8-10] |
|  |  |  | *Halictus scabiosae*  (Rossi, 1790) | 1 | 1 | [1-2-8-10] |
|  |  | *Lasioglossum* |  | 1 | 1 |  |
|  |  |  | *Lasioglossum albocinctum*  (Lucas, 1849) | 0 | 1 | [1-2-8-10] |
|  |  |  | *Lasioglossum nitidulum*  (Fabricius, 1804) | 1 | 0 | [1-2-8-10] |
|  |  | *Pseudapis* |  | 1 | 0 |  |
|  |  |  | *Pseudapis bispinosa*  (Brullé, 1832) | 1 | 0 | [2-8-10] |
|  |  | *Sphecodes* |  | 0 | 1 |  |
|  |  |  | *Sphecodes sf. ruficrus* | 0 | 1 |  |
|  | *Leucospidae* |  |  | 0 | 1 |  |
|  |  | *Leucospis* |  | 0 | 1 |  |
|  |  |  | *Leucospis*  *gigas*  (Fabricius, 1793) | 0 | 1 | - |
|  | *Megachilidae* |  |  | 1 | 1 |  |
|  |  | *Anthidium* |  | 0 | 1 |  |
|  |  |  | *Anthidium florentinum*  (Fabricius, 1775) | 0 | 1 | [1-2-10] |
|  |  |  |  |  |  |  |
|  |  |  |  |  |  |  |
|  |  | *Heriades* |  | 1 | 0 |  |
|  |  | *Megachile* |  | 0 | 1 |  |
|  |  |  | *Megachile sicula*  (Rossi, 1792) | 0 | 1 | [1-2-8-10] |
|  |  | *Osmia* |  | 1 | 0 |  |
|  |  |  | *Osmia*  *aurulenta*  (Panzer, 1799) | 1 | 0 | [1-2-8-10] |
|  |  |  | *Osmia*  *latreillei*  (Spinola, 1806) | 1 | 0 | [1-2-10] |
|  |  | *Rhodanthidium* |  | 1 | 1 |  |
|  |  |  | *Rhodanthidium septemdentatum*  (Latreille, 1809) | 1 | 1 | [1-2-8-10] |
|  |  |  | *Rhodanthidium sticticum*  (Fabricius, 1787) | 0 | 1 | [1-2-8-10] |
|  | *Pollistidae* |  |  | 1 | 1 |  |
|  |  | *Bembecinus* |  | 0 | 1 |  |
|  |  |  | *Bembecinus tridens*  (Fabricius, 1781) | 0 | 1 | [1-2-10] |
|  |  | *Polistes* |  | 1 | 1 |  |
|  |  |  | *Polistes dominula*  (Christ, 1791) | 1 | 1 | [1-2-8-9-10] |
|  |  |  | *Polistes*  *gallicus*  (Linnaeus, 1767) | 1 | 1 | [1-2-8-9-10] |
|  | *Pompilidae* |  |  | 1 | 1 |  |
|  |  | *Entomobora* |  | 1 | 1 |  |
|  |  |  | *Entomobora fuscipennis*  (Vander Linden, 1827) | 1 | 0 | [2-10] |
|  | *Scoliidae* |  |  | 1 | 1 |  |
|  |  | *Dasyscolia* |  | 0 | 1 |  |
|  |  |  | *Dasyscolia ciliata*  (Fabricius, 1787) | 0 | 1 | [1-2-8-10] |
|  |  | *Megascolia* |  | 1 | 1 |  |
|  |  |  | *Megascolia bidens*  (Linnaeus, 1767) | 1 | 1 | [1-2-8-9-10] |
|  |  |  | *Megascolia maculata*  (Drury, 1773) | 1 | 1 | [1-2-8-9-10] |
|  | *Sphecidae* |  |  | 0 | 1 |  |
|  |  | *Sphex* |  | 0 | 1 |  |
|  |  |  | *Sphex*  *funerarius*  (Gussakovskij, 1934) | 0 | 1 | [1-2-8-10] |
|  | *Syrphidae* |  |  | 1 | 1 |  |
|  |  | *Sphaerophoria* |  | 1 | 1 |  |
|  | *Typhiidae* |  |  | 1 | 0 |  |
|  |  | *Meria* |  | 1 | 0 |  |
|  |  |  | *Meria*  *tripunctata*  (Rossi, 1790) | 1 | 0 | [1-2-8-10] |
|  | *Vespidae* |  |  | 1 | 1 |  |
|  |  | *Ancistrocerus* |  | 0 | 1 |  |
|  |  |  | *Ancistrocerus kitcheneri*  (Dusmet, 1917) | 0 | 1 | [1-2-8-10] |
|  |  | *Eumenes* |  | 1 | 0 |  |
|  |  |  | *Eumenes coarctatus*  (Linnaeus, 1758) | 1 | 0 | [1-2-8-10] |
| *Lepidoptera* |  |  |  | 1 | 1 |  |
|  | *Lycaenidae* |  |  | 1 | 1 |  |
|  |  | *Celastrina* |  | 0 | 1 |  |
|  |  |  | *Celastrina argiolus*  (Linnaeus, 1758) | 0 | 1 | [1-8-9-10] |
|  |  | *Polyommatus* |  | 1 | 1 |  |
|  |  |  | *Polyommatus celina*  (Austaut, 1879) | 1 | 1 | [1-8-9-10] |
|  | *Noctuidae* |  |  | 0 | 1 |  |
|  |  | *Autographa* |  | 0 | 1 |  |
|  |  |  | *Autographa gamma*  (Linnaeus, 1758) | 0 | 1 | [1-8-9-10] |
|  |  | *Odice* |  | 0 | 1 |  |
|  |  |  | *Odice*  *blandula*  (Rambur, 1858) | 0 | 1 | 10 |
|  | *Nymphalidae* |  |  | 1 | 1 |  |
|  |  | *Vanessa* |  | 1 | 1 |  |
|  |  |  | *Vanessa*  *atalanta*  (Linnaeus, 1758) | 0 | 1 | [1-8-9-10] |
|  |  |  | *Vanessa*  *cardui*  (Linnaeus, 1758) | 1 | 1 | [1-8-9-10] |
|  | *Pieridae* |  |  | 1 | 1 |  |
|  |  | *Colias* |  | 1 | 1 |  |
|  |  |  | *Colias*  *croceus*  (Fourcroy, 1785) | 1 | 1 | [1-8-9-10] |
|  |  | *Gonepteryx* |  | 0 | 1 |  |
|  |  |  | *Gonepteryx cleopatra*  (Linnaeus, 1767) | 0 | 1 | [1-8-9-10] |
|  |  | *Pieris* |  | 1 | 1 |  |
|  |  |  | *Pieris*  *rapae*  (Linnaeus, 1758) | 1 | 1 | [1-8-9-10] |
|  |  | *Pontia* |  | 1 | 0 |  |
|  |  |  | *Pontia*  *daplidice*  (Linaneus, 1758) | 1 | 0 | [1-8-9-10] |
|  | *Satyridae* |  |  | 0 | 1 |  |
|  |  | *Pararge* |  | 0 | 1 |  |
|  |  |  | *Pararge*  *aegeria*  (Linnaeus, 1758) | 0 | 1 | [1-8-9-10] |

**Table S3**. Pollinator species metrics of the different populations (Son Serra, Es Trenc) And both (All). *D*= Number of different plant species visited, *Spec*= Specificity. Selectiveness is calculated based on the *Specificity index* (highly selective, *Spec* > 0.75; selective, 0.75 > *Spec* > 0.5; opportunistic, 0.5 > *Spec* > 0.25; highly opportunistic, *Spec* < 0.25).

|  |  |  |  |  |  |  |  |
| --- | --- | --- | --- | --- | --- | --- | --- |
|  | Es Trenc | | Son Serra | | All | | |
| Species | *D* | *Spec* | *D* | *Spec* | *D* | *Spec* | *Specificity* |
| *Amegilla albigena* | 1 | 1 |  |  | 1 | 1 | Highly selective |
| *Amegilla quadrifasciata* |  |  | 3 | 0,53 | 3 | 0,53 | Selective |
| *Ancistrocerus kitcheneri* |  |  | 1 | 1 | 1 | 1 | Highly selective |
| *Andrena agilissima* |  |  | 1 | 1 | 1 | 1 | Highly selective |
| *Andrena flavipes* | 1 | 1 |  |  | 1 | 1 | Highly selective |
| *Andrena morio* | 1 | 1 | 5 | 0,37 | 5 | 0,38 | Opportunistic |
| *Anthaxia umbellatarum* | 1 | 1 |  |  | 1 | 1 | Highly selective |
| *Anthidium florentinum* |  |  | 1 | 1 | 1 | 1 | Highly selective |
| *Apis mellifera* | 3 | 0,47 | 10 | 0,18 | 10 | 0,19 | Highly opportunistic |
| *Autographa gamma* |  |  | 1 | 1 | 1 | 1 | Highly selective |
| *Bembecinus tridens* |  |  | 1 | 1 | 1 | 1 | Highly selective |
| *Bombus terrestris* |  |  | 5 | 0,37 | 5 | 0,38 | Opportunistic |
| *Celastrina argiolus* |  |  | 1 | 1 | 1 | 1 | Highly selective |
| *Ceratina cucurbitina* | 1 | 1 |  |  | 1 | 1 | Highly selective |
| *Cerceris arenaria* | 1 | 1 |  |  | 1 | 1 | Highly selective |
| *Cerceris sabulosa* | 1 | 1 |  |  | 1 | 1 | Highly selective |
| *Ceylalictus variegatus* | 1 | 1 |  |  | 1 | 1 | Highly selective |
| *Megachile sicula* |  |  | 1 | 1 | 1 | 1 | Highly selective |
| *Coccinella septempunctata* | 1 | 1 | 1 | 1 | 1 | 1 | Highly selective |
| *Colias croceus* | 1 | 1 | 1 | 1 | 1 | 1 | Highly selective |
| *Colletes abeillei* | 3 | 0,47 | 2 | 0,68 | 4 | 0,44 | Opportunistic |
| *Colletes succinctus* | 1 | 1 |  |  | 1 | 1 | Highly selective |
| *Dasyscolia ciliata* |  |  | 2 | 0,68 | 2 | 0,68 | Selective |
| *Dipogon variegatus* |  |  | 1 | 1 | 1 | 1 | Highly selective |
| *Entomobora fuscipennis* | 1 | 1 |  |  | 1 | 1 | Highly selective |
| *Eristalinus aeneus* | 1 | 1 | 4 | 0,44 | 4 | 0,44 | Opportunistic |
| *Eristalinus sepulchralis* |  |  | 2 | 0,68 | 2 | 0,68 | Selective |
| *Eristalis tenax* |  |  | 3 | 0,53 | 3 | 0,53 | Selective |
| *Eumenes coarctatus* | 1 | 1 |  |  | 1 | 1 | Highly selective |
| *Exoprosopa italica* | 1 | 1 | 2 | 0,68 | 3 | 0,53 | Selective |
| *Gonepteryx cleopatra* |  |  | 4 | 0,44 | 4 | 0,44 | Opportunistic |
| *Graphosoma lineatum* | 1 | 1 |  |  | 1 | 1 | Highly selective |
| *Halictus fulvipes* |  |  | 1 | 1 | 1 | 1 | Highly selective |
| *Halictus scabiosae* | 1 | 1 | 1 | 1 | 1 | 1 | Highly selective |
| *Hylaeus annularis* |  |  | 1 | 1 | 1 | 1 | Highly selective |
| *Hylaeus pictus* | 1 | 1 | 1 | 1 | 2 | 0,68 | Selective |
| *Lasioglossum albocinctum* |  |  | 2 | 0,68 | 2 | 0,68 | Selective |
| *Lasioglossum nitidulum* | 1 | 1 |  |  | 1 | 1 | Highly selective |
| *Leucospis gigas* |  |  | 1 | 1 | 1 | 1 | Highly selective |
| *Lucilia sericata* | 1 | 1 |  |  | 1 | 1 | Highly selective |
| *Megascolia bidens* | 3 | 0,47 | 2 | 0,68 | 3 | 0,53 | Selective |
| *Megascolia maculata* | 2 | 0,65 | 2 | 0,68 | 3 | 0,53 | Selective |
| *Melecta luctuosa* |  |  | 1 | 1 | 1 | 1 | Highly selective |
| *Meria tripunctata* | 1 | 1 |  |  | 1 | 1 | Highly selective |
| *Myopa extricata* |  |  | 1 | 1 | 1 | 1 | Highly selective |
| *Nomada succinta* | 1 | 1 |  |  | 1 | 1 | Highly selective |
| *Oedemera flavipes* |  |  | 1 | 1 | 1 | 1 | Highly selective |
| *Osmia aurulenta* | 1 | 1 |  |  | 1 | 1 | Highly selective |
| *Osmia latreillei* | 1 | 1 |  |  | 1 | 1 | Highly selective |
| *Oxythyrea funesta* |  |  | 5 | 0,37 | 5 | 0,38 | Opportunistic |
| *Paragus haemorrhous* |  |  | 1 | 1 | 1 | 1 | Highly selective |
| *Pararge aegeria* |  |  | 1 | 1 | 1 | 1 | Highly selective |
| *Philanthus triangulum* | 1 | 1 | 3 | 0,53 | 3 | 0,53 | Selective |
| *Pieris rapae* | 1 | 1 | 2 | 0,68 | 3 | 0,53 | Selective |
| *Pollistes dominula* | 1 | 1 | 2 | 0,68 | 2 | 0,68 | Selective |
| *Pollistes gallicus* | 1 | 1 | 2 | 0,72 | 3 | 0,57 | Selective |
| *Polyommatus celina* | 5 | 0,26 | 4 | 0,44 | 6 | 0,33 | Opportunistic |
| *Pontia daplidice* | 1 | 1 |  |  | 1 | 1 | Highly selective |
| *Pseudapis bispinosa* | 2 | 0,65 |  |  | 2 | 0,68 | Selective |
| *Sphaerophoria rueppellii* |  |  | 1 | 1 | 1 | 1 | Highly selective |
| *Sphaerophoria taeniata* | 1 | 1 |  |  | 1 | 1 | Highly selective |
| *Sphex funerarius* |  |  | 2 | 0,68 | 2 | 0,68 | Selective |
| *Stizus fasciatus* |  |  | 1 | 1 | 1 | 1 | Highly selective |
| *Stomorhina lunata* |  |  | 2 | 0,68 | 2 | 0,68 | Selective |
| *Syritta pipiens* | 1 | 1 |  |  | 1 | 1 | Highly selective |
| *Thaumatomyia notata* | 1 | 1 |  |  | 1 | 1 | Highly selective |
| *Tropinota squalida* |  |  | 1 | 1 | 1 | 1 | Highly selective |
| *Vanessa atalanta* |  |  | 1 | 1 | 1 | 1 | Highly selective |
| *Vanessa cardui* | 2 | 0,65 | 2 | 0,68 | 3 | 0,53 | Selective |
| *Xylocopa violacea* |  |  | 1 | 1 | 1 | 1 | Highly selective |

**Table S4**. Pollinator family metrics. *D*= Number of different plant species visited, *Spec*= Specificity. Selectiveness is calculated based on the *Specificity index* (highly selective, *Spec* > 0.75; selective, 0.75 > *Spec* > 0.5; opportunistic, 0.5 > *Spec* > 0.25; highly opportunistic, *Spec* < 0.25).

| *Family* | *Number_species* | *Number_plants* | *Spec* | *Specificity* |
| --- | --- | --- | --- | --- |
| Andrenidae | 4 | 5 | 0.38 | Opportunistic |
| Apidae | 9 | 11 | 0.17 | Highly opportunistic |
| Bombyllidae | 2 | 3 | 0.54 | Selective |
| Bruchidae | 1 | 1 | 1.00 | Highly selective |
| Buprestidae | 2 | 1 | 1.00 | Highly selective |
| Calliphoridae | 3 | 2 | 0.68 | Selective |
| Cloropidae | 1 | 1 | 1.00 | Highly selective |
| Coccinellidae | 2 | 1 | 1.00 | Highly selective |
| Colletidae | 7 | 4 | 0.45 | Opportunistic |
| Conopidae | 2 | 3 | 0.54 | Selective |
| Crabronidae | 5 | 3 | 0.54 | Selective |
| Ephydriidae | 1 | 1 | 1.00 | Highly selective |
| Halictidae | 8 | 7 | 0.29 | Opportunistic |
| Leucospidae | 1 | 1 | 1.00 | Highly selective |
| Lycaenidae | 2 | 6 | 0.33 | Opportunistic |
| Megachilidae | 7 | 2 | 0.68 | Selective |
| Mordellidae | 1 | 1 | 1.00 | Highly selective |
| Muscidae | 1 | 1 | 1.00 | Highly selective |
| Noctuidae | 3 | 1 | 1.00 | Highly selective |
| Nymphalidae | 2 | 3 | 0.54 | Selective |
| Oedemerinae | 1 | 1 | 1.00 | Highly selective |
| Pentatomidae | 1 | 1 | 1.00 | Highly selective |
| Phoridae | 1 | 1 | 1.00 | Highly selective |
| Pieridae | 4 | 8 | 0.26 | Opportunistic |
| Polistidae | 4 | 3 | 0.54 | Selective |
| Polistinae | 3 | 1 | 1.00 | Highly selective |
| Pollistidae | 1 | 1 | 1.00 | Highly selective |
| Pompilidae | 3 | 1 | 1.00 | Highly selective |
| Sarcophagidae | 1 | 1 | 1.00 | Highly selective |
| Satyridae | 1 | 1 | 1.00 | Highly selective |
| Scarabaeidae | 2 | 6 | 0.33 | Opportunistic |
| Scoliidae | 4 | 3 | 0.54 | Selective |
| Scraptiidae | 1 | 1 | 1.00 | Highly selective |
| Sphecidae | 1 | 2 | 0.68 | Selective |
| Syrphidae | 9 | 6 | 0.33 | Opportunistic |
| Typhiidae | 1 | 1 | 1.00 | Highly selective |
| Vespidae | 3 | 1 | 1.00 | Highly selective |

**Table S5**. Pollinator order metrics of the different populations. *D*= Number of different plant species visited, *Spec*= Specificity. Selectiveness is calculated based on the *Specificity index* (highly selective, *Spec* > 0.75; selective, 0.75 > *Spec* > 0.5; opportunistic, 0.5 > *Spec* > 0.25; highly opportunistic, *Spec* < 0.25).

| *Orden* | *Number of species* | *Number of plants* | *Spec* | *Specific* |
| --- | --- | --- | --- | --- |
| Coleoptera | 9 | 6 | 0.33 | Opportunistic |
| Diptera | 17 | 9 | 0.23 | Highly opportunistic |
| Hymenoptera | 47 | 15 | 0.07 | Highly opportunistic |
| Lepidoptera | 12 | 9 | 0.23 | Highly opportunistic |

**Table S6**. Pollinator behaviour results of the focal census on *E. maritimum* individuals. Time spent in visits and number of visited capitula are indicated. For some species only one observation is available.

| ***Order*** | ***Family*** | ***Genus*** | ***Species*** | ***Time (s)*** | ***Capitula*** |
| --- | --- | --- | --- | --- | --- |
| *Coleoptera* |  |  |  |  | 2 |
| *Diptera* |  |  |  | 67 ± 2.35 | 1 |
|  | *Calliphoridae* |  |  | 2.5 ± 0.5 | 1 |
| *Diptera* | *Syrphidae* |  |  | 3 ± 0 | 4 |
| *Hymenoptera* |  |  |  | 81.45 ± 21.41 | 3 ± 0.48 |
|  | *Apidae* | *Amegilla* | *Amegilla quadrifasciata* | 5.5 ± 0.5 | 5 |
|  |  | *Bombus* | *Bombus*  *terrestris* | 36.78 ± 14.62 | 5.67 ± 2.4 |
|  |  | *Pseudapis* | *Pseudapis bispinosa* | 30 | 2 |
|  | *Crabronidae* |  |  | 74 | 3 |
|  |  | *Cerceris* |  | 14 | 1 |
|  |  |  | *Cerceris*  *arenaria* | 12 | 1 |
|  |  | *Philanthus* | *Philanthus triangulum* | 117 ± 23 |  |
|  | *Colletidae* |  |  | 10 ± 4 | 1.5 ± 0.5 |
|  |  | *Colletes* | *Colletes*  *succintus* | 14 | 2 |
|  | *Halictidae* |  |  | 14 | 1 |
|  |  | *Ceratina* | *Ceratina cucurbitina* | 20 ± 14 | 1 |
|  |  | *Halictus* | *Halictus scabiosae* | 28.4 ± 7.54 | 1.2 ± 0.2 |
|  | *Polistidae* |  |  | 13 | 1 |
|  |  | *Polistes* | *Pollistes dominula* | 16.67 ± 7.69 | 1 |
|  |  |  | *Pollistes*  *gallicus* | 11 | 1 |
| *Hymenoptera* | *Pompilidae* | *Entomobora* | *Entomobora fuscipennis* | 10 | 1 |
|  | *Scoliidae* |  |  | 197 ± 14 | 6 |
|  |  | *Dasyscolia* | *Dasyscolia*  *ciliata* | 65 | 4 |
|  |  | *Megascolia* | *Megascolia bidens* | 32.67 ± 9.4 | 2.33 ± 0.88 |
|  |  |  | *Megascolia maculata* | 32.75 ± 9.7 | 1.67 ± 0.67 |
|  | *Sphecidae* | *Sphex* | *Sphex funerarius* | 20 | 3 |
| *Lepidoptera* |  |  |  |  |  |

REFERENCES:

Baldock, D. W., Livory, A., & Owens, N. W. (2020). The bees and wasps of the Balearic Islands (Hymenoptera: Chrysidoidea, Vespoidea, Apoidea) with a discussion of aculeate diversity and endemism in Mediterranean and Atlantic archipelagos. Entomofauna (Supplement), 25, 1–202.

Cobos, A. (1986). Fauna iberica de coleopteros Buprestidae. Editorial CSIC-CSIC Press.

Ebejer, M. J. (2006). Some Chloropidae (Diptera) from the Balearic Islands (Spain) with particular reference to parc natural de s' Albufera de Mallorca. Alguns Chloropidae (Diptera) de les Illes Balears (Espanya) amb especial referencia al parc natural de s' Albufera de …. Bolletí de la Societat d'Història Natural de les Balears, 49,173–184.

Gomila, D. G. (1999). Uso recreativo en los espacios naturales de Mallorca: el área natural de especial interés (ANEI) de Es Carnatge d'es Coll den Rabassa. Papeles de Geografía, 3, 47–65.

Tolrà, M.C. (2002). Catálogo de los Diptera de España, Portugal y Andorra. Sociedad Entomológica Aragonesa.

Vives, A. (1994). Catálogo sistemático y sinonímico de los lepidopteros de la Península Ibérica y Baleares. Ministerio de Agricultura, Pesca y Alimentación.
